# The pivotal role of the Hes1/Piezo1 pathway in the pathophysiology of glucocorticoid-induced osteoporosis

**DOI:** 10.1172/jci.insight.179963

**Published:** 2024-12-06

**Authors:** Nagahiro Ochiai, Yuki Etani, Takaaki Noguchi, Taihei Miura, Takuya Kurihara, Yuji Fukuda, Hidetoshi Hamada, Keisuke Uemura, Kazuma Takashima, Masashi Tamaki, Teruya Ishibashi, Shohei Ito, Satoshi Yamakawa, Takashi Kanamoto, Seiji Okada, Ken Nakata, Kosuke Ebina

**Affiliations:** 1 Department of Musculoskeletal Regenerative Medicine, Graduate School of Medicine, Osaka University, Suita, Osaka, Japan.; 2 Taisho Pharmaceutical Co., Ltd., Saitama, Japan.; 3 Department of Orthopaedic Surgery,; 4 Department of Orthopaedic Medical Engineering, and; 5 Department of Orthopaedic Biomaterial Science, Graduate School of Medicine, Osaka University, Suita, Osaka, Japan.; 6 Department of Sports Medical Biomechanics, and; 7 Department of Health and Sport Sciences, Osaka University Graduate School of Medicine, Suita, Osaka, Japan.

**Keywords:** Bone biology, Therapeutics, Bone disease, Osteoporosis

## Abstract

Glucocorticoid-induced osteoporosis (GIOP) lacks fully effective treatments. This study investigated the role of Piezo1, a mechanosensitive ion channel component 1, in GIOP. We found reduced Piezo1 expression in cortical bone osteocytes from patients with GIOP and a GIOP mouse model. Yoda1, a Piezo1 agonist, enhanced the mechanical stress response and bone mass and strength, which were diminished by dexamethasone (DEX) administration in GIOP mice. RNA-seq revealed that Yoda1 elevated Piezo1 expression by activating the key transcription factor Hes1, followed by enhanced CaM kinase II and Akt phosphorylation in osteocytes. This improved the lacuno-canalicular network and reduced sclerostin production and the receptor activator of NF-κB/osteoprotegerin ratio, which were mitigated by DEX. Comparative analysis of mouse models and human GIOP cortical bone revealed downregulation of mechanostimulated osteogenic factors, such as osteocrin, and cartilage differentiation markers in osteoprogenitor cells. In human periosteum-derived cells, DEX suppressed differentiation into osteoblasts, but Yoda1 rescued this effect. Our findings suggest that reduced Piezo1 expression and activity in osteocytes and periosteal cells contribute to GIOP, and Yoda1 may offer a novel therapeutic approach by restoring mechanosensitivity.

## Abstract 2

In glucocorticoid-induced osteoporosis, transcription factor Hes1 and mechanosensitive ion channel Piezo1 play central roles, and a Piezo1 activator Yoda1 ameliorates the pathology

## Graphical abstract

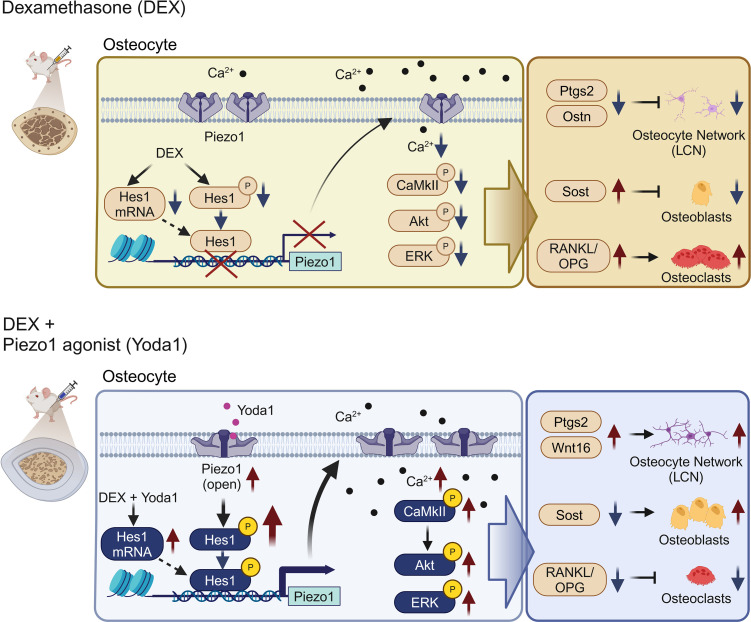

## Introduction

Glucocorticoid-induced osteoporosis (GIOP) is a major side effect observed in patients with autoimmune disease who receive glucocorticoids (1, 2). Bone loss reaches its peak within the first 3 to 6 months of treatment due to increased osteoclast activity and elevated osteoblast and osteocyte apoptosis (1, 2). Fracture risk in GIOP is correlated with bone mineral density (BMD) to a lesser degree than in traditional osteoporosis, suggesting that glucocorticoids have a negative impact on bone quality and mass (1–3). In GIOP, a decrease in the osteocyte lacuno-canaliculi network (LCN) has been observed, but the molecular basis of this phenomenon remains poorly understood (4). The LCN is a fluid-filled framework that permeates bones and houses an osteocyte network (5, 6). It is believed that mechanosensation arises from the cellular detection of forces generated by load-induced fluid flow within this structure (5, 6). Importantly, mechanical stress exercises have been shown to enhance BMD in GIOP (7, 8). Although alendronate alone is insufficient, its combination with mechanical stress significantly boosts BMD (8). These findings suggest that treatments mimicking mechanical stress on osteocytes could be a breakthrough in GIOP drug development.

Osteocytes play a crucial role in bone remodeling by coordinating the activities of osteoblasts and osteoclasts through the regulation of factors such as sclerostin (Sost), receptor activator of NF-κB ligand (RANKL), osteoprotegerin (OPG), and other signaling molecules within the osteocyte network (9, 10). Among these factors, the mechanosensitive channel Piezo1 has garnered significant attention for its role in the osteocyte response to mechanical stimuli (11–16). Studies using conditional osteocyte-specific Piezo1-knockout mice have demonstrated that the absence of Piezo1 leads to compromised cortical bone parameters, highlighting the importance of Piezo1 in mediating mechanically induced bone formation through calcium influx and the upregulation of the Wnt pathway (11, 13, 14). However, the precise molecular mechanisms underlying Piezo1-related signaling and its transcriptional regulation are yet to be fully clarified.

Yoda1, a Piezo1 agonist identified through high-throughput screening, has been shown to increase BMD in intact rodents and reduce the healing time of bone fractures in mice (13, 17, 18). Li et al. reported that Yoda1 promotes the differentiation of mouse periosteum-derived cells (PDCs) into osteoblasts and accelerates fracture healing in a tail suspension model, suggesting an enhancement of mechanoresponsive pathways through Piezo1 activation (18). However, the impact of Piezo1 activation on mechanical stress in GIOP mouse models during disease progression has not been reported. We hypothesized that GIOP mice have impaired responses to mechanical stress and that Yoda1, a Piezo1 activator, may restore the response to mechanical stress in GIOP models. The aim of this study was to elucidate the role and mechanism of the mechanosensitive receptor Piezo1 in the pathogenesis of GIOP and to examine whether activation of the Piezo1 pathway can ameliorate the condition of GIOP. We conducted histological analysis of the femoral neck cortical bone in patients with GIOP and non-GIOP volunteers, investigated the effects of Yoda1 administration in an s.c. dexamethasone-injected (DEX-injected) GIOP mouse model and mechanical loading model of the tibia, and performed RNA-seq analysis of cortical bone from humans and mice with and without GIOP.

## Results

### Patients with GIOP demonstrate a decrease in both the lacunae-osteocyte network and Piezo1 protein expression.

**Figure 1:**
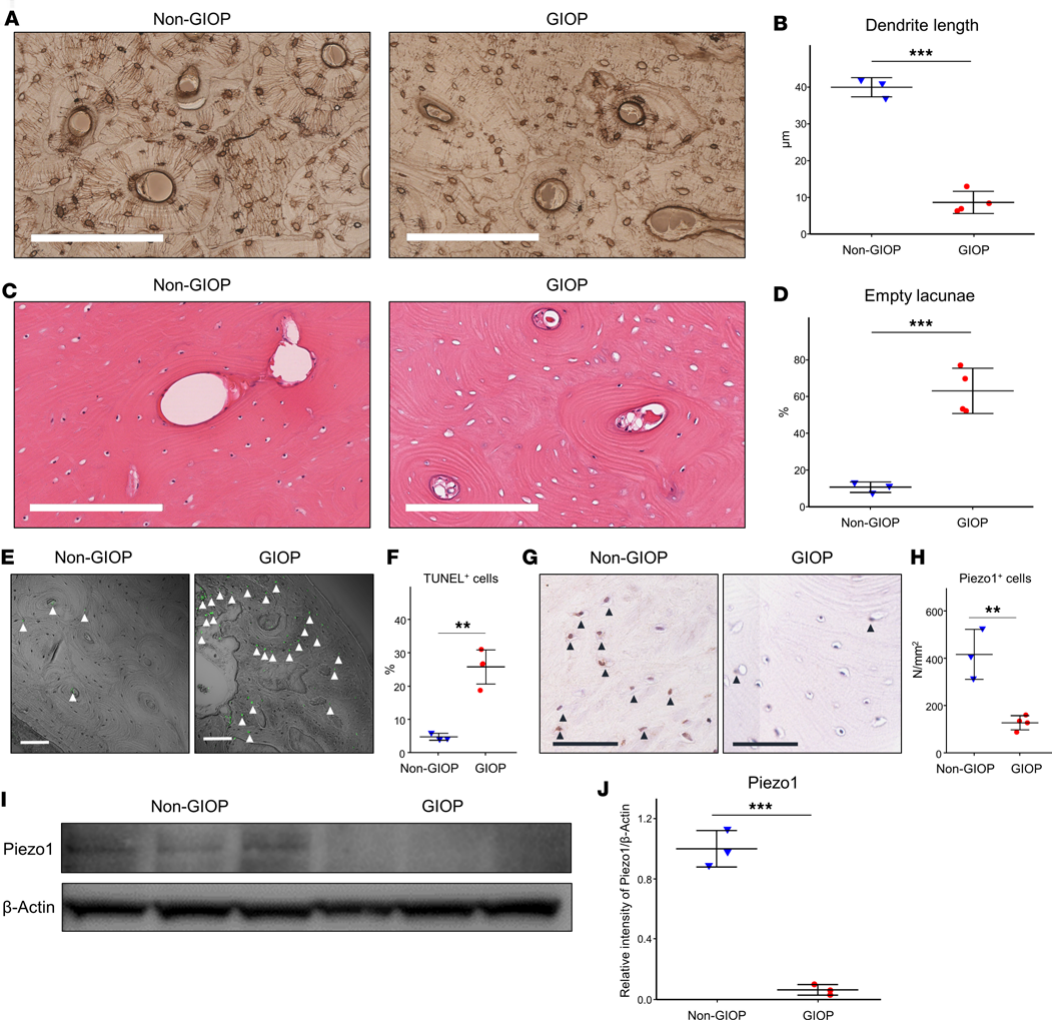
Patients with GIOP exhibit a reduced osteocyte LCN and Piezo1 protein expression. The figure depicts cortical bone sections from the femurs of both patients with GIOP and non-GIOP controls (Supplemental Table 1). (**A** and **B**) Silver staining, highlighting the lacunae-osteocyte network. (**A**) Comparison between a non-GIOP control (52 years, female) and a patient with GIOP (51 years, female). Original magnification, ×4; scale bars: 200 μm. (**B**) Quantification of the dendrite length of osteocytes in the 2 groups. (**C** and **D**) Hematoxylin and eosin (H&E) staining. Images of sections from a non-GIOP control (52 years, female) and a patient with GIOP (51 years, female). Original magnification, ×4; scale bars: 200 μm. (**D**) The ratio of empty lacunae to total lacunae was calculated. (**E** and **F**) TUNEL assay results, with images (**E**) comparing a non-GIOP control (61 years, female) with a patient with GIOP (51 years, female). Original magnification, ×20; scale bars: 100 μm. (**F**) Quantification of TUNEL^+^ cells. (**G** and **H**) IHC analysis showcasing Piezo1 expression. (**G**) Comparison between a non-GIOP control (65 years, female) and a patient with GIOP (63 years, female). Original magnification,× 40; scale bars: 60 μm. (**H**) Quantification of Piezo1^+^ cells per bone surface area. (**I**) WB results for Piezo1 and β-actin. Data are expressed as mean ± SD, *n* = 3 per group. For WB analysis of Piezo1 and β-actin protein expression, results are expressed as mean ± SD. (**J**) Quantification of WB analysis using ImageJ. Band intensities were normalized to β-actin. A 2-tailed Student’s *t* test with a 95% confidence interval was used for statistical analysis. **P < 0.01, ***P < 0.001.

To investigate the pathophysiological differences between GIOP and non-GIOP bone tissues, femoral neck samples were harvested during total hip arthroplasty, and the calcar regions from these specimens were examined (Figure 1 and Supplemental Table 1; supplemental material available online with this article; https://doi.org/10.1172/jci.insight.179963DS1). The osteocyte LCN was visualized using silver staining, revealing a significant reduction in dendritic length in patients with GIOP compared with their non-GIOP counterparts (Figure 1, A and B). The ratio of empty lacunae was also significantly higher in GIOP than in non-GIOP tissues (Figure 1, C and D). Furthermore, a notable increase in TUNEL-positive osteocytes was observed in the GIOP group (Figure 1, E and F). Remarkably, IHC and Western blot (WB) analyses revealed downregulation of Piezo1 in the osteocytes of patients with GIOP (Figure 1, G–J). Conversely, the quantity of tartrate-resistant acid phosphatase–positive (TRAP-positive) osteoclasts did not significantly differ between GIOP and non-GIOP bone tissues (Supplemental Figure 1).

### Yoda1 prevents bone structure changes and fragility in the femur of a GIOP mouse model.

**Figure 2:**
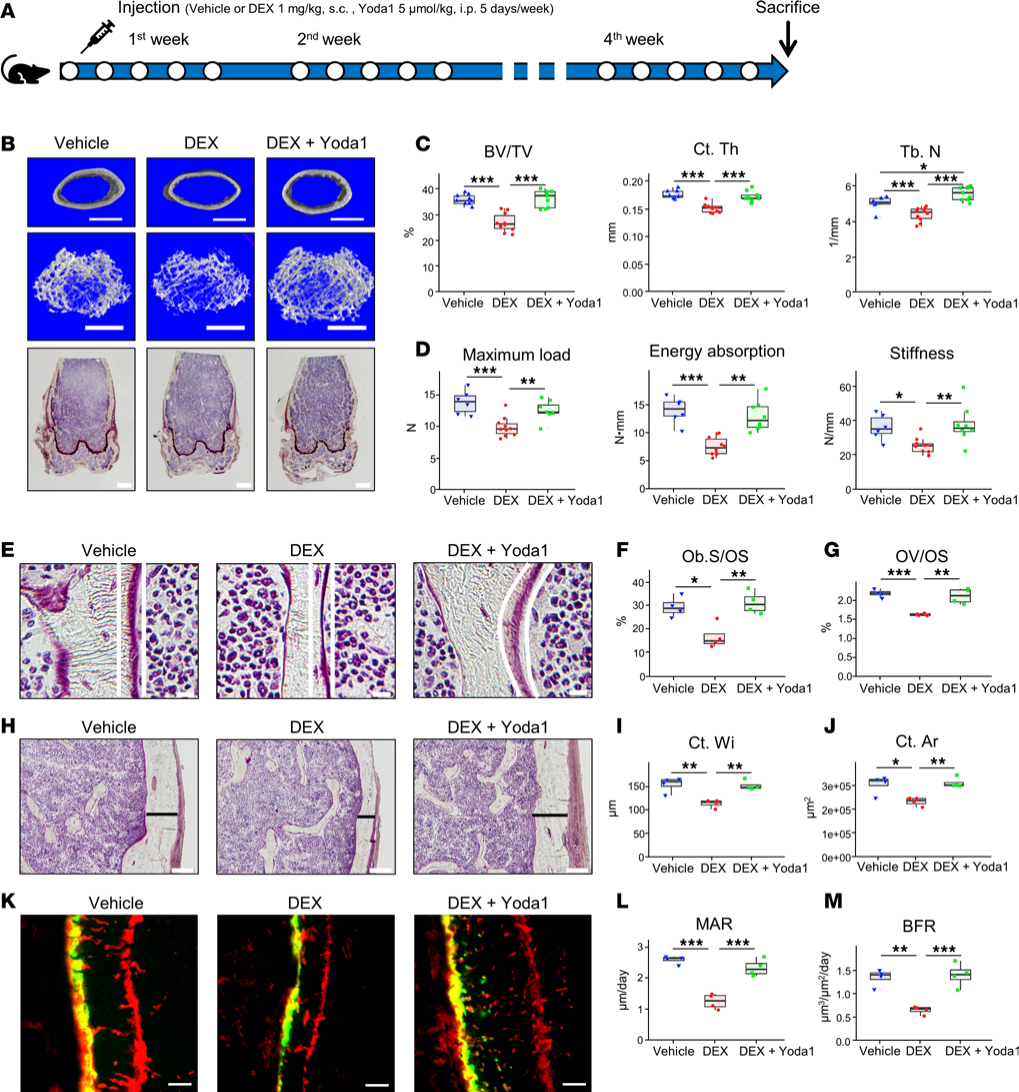
Yoda1 prevents bone structure changes and reduces the fragility of the femur in a mouse model of GIOP. (**A**) Injection schedule for DEX and Yoda1: DEX was administered 1 mg/kg (s.c.), while Yoda1 was injected 5 μmol/kg (i.p.), and vehicle treatments consisting of distilled water was injected s.c. like DEX and 5% ethanol was injected i.p. like Yoda1. Each group received 5 injections over 1 week, repeated for 4 weeks. Experimental groups were defined as Vehicle (vehicle-treated), DEX (DEX-treated), and DEX+Yoda1 (concomitant administration of DEX and Yoda1). (**B**) Microcomputed tomography images (top 2 rows) of cortical and trabecular bone (scale bars: 250 μm) and femur metaphysis area (bottom row) stained using Villanueva’s bone staining (scale bars: 100 μm). (**C**) Microcomputed tomography analysis, including bone volume fraction (BV/TV), cortical thickness (Ct. Th.), and trabecular number (Tb. N.) (*n* = 9 for each group). (**D**) Three-point bending tests on femurs, with sample sizes as follows: Vehicle (*n* = 6), DEX (*n* = 10), and DEX+Yoda1 (*n* = 8). (**E**) Images highlighting the osteoid surface (indicated by white lines) at ×80 magnification during bone morphometry analysis (scale bars: 10 μm). (**F**) Osteoblast surface (Ob. S/OS). (**G**) Osteoid volume (OV/OS). (**H**) Cortical bone images (depicted with black bands) at ×80 magnification (scale bars: 10 μm). (**I**) Cortical width (Ct. Wi.). (**J**) Cortical area (Ct. Ar.). (**K**) Images of alizarin and calcein labeling at ×100 magnification (scale bars: 5 μm). The labeling periods were 4 days for alizarin and 1 day for calcein. (**L**) MAR. (**M**) BFR. For panels **E**–**M**, *n* = 4 for each group. The results are presented as box-and-whisker plots. Statistical significance was determined using a 1-way ANOVA followed by a Tukey-Kramer post hoc test. *P < 0.05, **P < 0.01, ***P < 0.001.

The effects of Yoda1, a Piezo1-specific agonist, were investigated in the GIOP model (Figure 2). Mice were treated with a concurrent administration of DEX and Yoda1 for 4 weeks, and the femoral microarchitecture was assessed (Figure 2A). Microcomputed tomography analysis revealed a significant reduction in bone volume fraction (BV/TV), trabecular number (Tb. N.), and cortical thickness (Ct. Th.) in the DEX-treated group compared with the vehicle-treated group. Notably, these decreases were almost completely mitigated in the group treated with a combination of DEX and Yoda1 (Figure 2, B and C). The cortical bone porosity (Po. tot and Po. V) and parameters associated with the trabecular bone structure were significantly exacerbated in the DEX-treated group, but were attenuated by Yoda1 administration (Supplemental Figure 2). The mechanical 3-point bending test revealed that the DEX-treated group exhibited significantly lower maximum load, energy absorption, and stiffness than the vehicle-treated group. However, these parameter changes were prevented by Yoda1 administration (Figure 2D). Bone histomorphometric analyses revealed notable decreases in osteoblast surface (Ob. S/OS), osteoid volume (OV/OS), cortical width (Ct. Wi.), and cortical area (Ct. Ar.) in the DEX-treated group. In contrast, treatment with Yoda1 effectively prevented these decreases (Figure 2, E–J, and Supplemental Figure 3). Furthermore, DEX treatment led to a significant reduction in mineral apposition rate (MAR) and bone formation rate (BFR), whereas these parameters were maintained even with DEX treatment in the Yoda1-treated group (Figure 2, K–M). The bone mass increase observed with Yoda1 treatment alone was not as pronounced as when Yoda1 was administered to the GIOP model mouse (Supplemental Figure 4).

**Figure 3:**
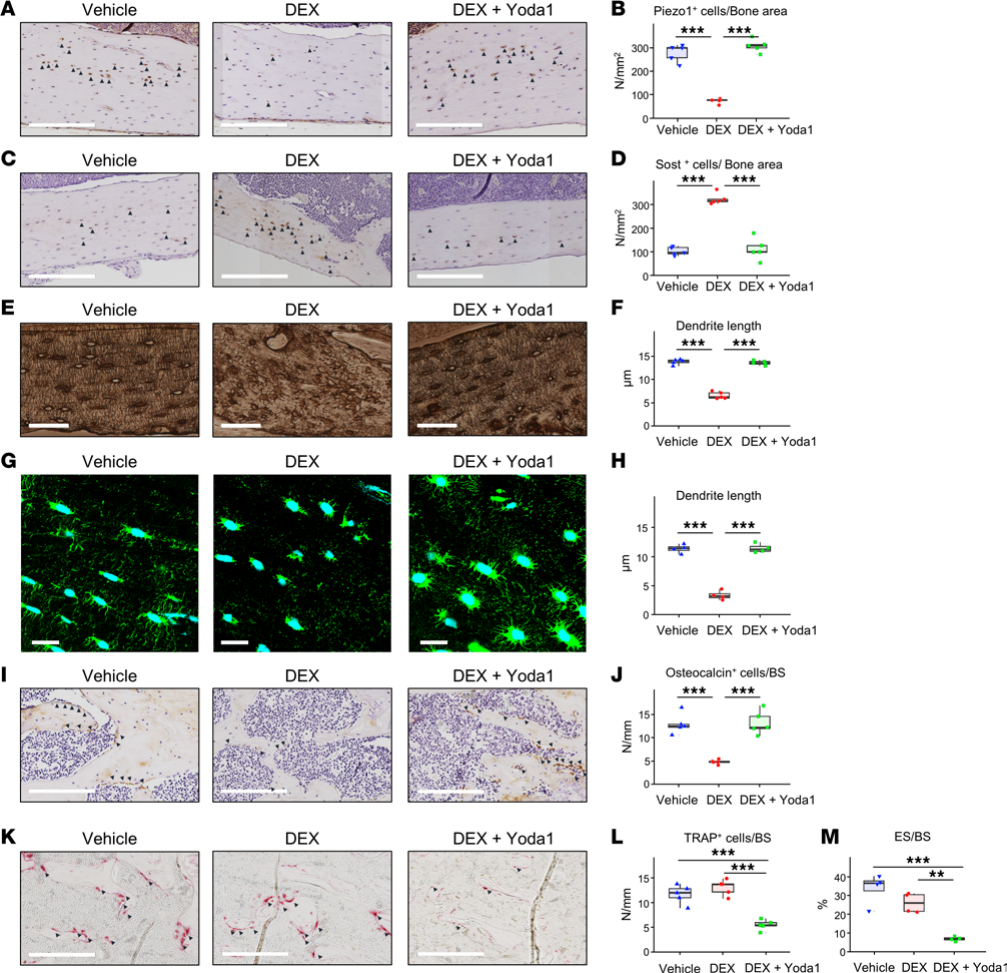
Bone histological analysis following combination treatment with DEX and Yoda1. Experimental groups consisted of Vehicle (treated with distilled water s.c. and 5% ethanol i.p.), DEX (treated with DEX, 1 mg/kg, s.c.), and DEX+Yoda1 (received concurrent treatment of DEX, 1 mg/kg, s.c. and Yoda1, 5 μmol/kg, i.p.). (**A** and **B**) IHC for Piezo1. (**A**) Piezo1^+^ cells are identified by arrowheads at ×20 magnification (scale bars: 200 μm). (**B**) Quantitative analysis of Piezo1^+^ cells per bone surface area. (**C** and **D**) IHC analysis of Sost. (**C**) Sost^+^ cells are denoted by arrowheads at ×20 magnification (scale bars: 200 μm). (**D**) Quantification of Sost^+^ cells per bone surface area. (**E** and **F**) Silver staining for LCN visualization. (**E**) LCN imaged at ×40 magnification (scale bars: 60 μm). (**F**) Quantification of the dendritic length of osteocytes across groups. (**G** and **H**) F-actin visualization with Alexa Fluor 488–labeled phalloidin staining, with nuclei counterstaining with DAPI. (**G**) Staining displayed at ×100 magnification (scale bars: 20 μm). (**H**) Quantification of osteocyte dendrite length among the groups. (**I** and **J**) IHC for osteocalcin. (**I**) Osteocalcin^+^ cells indicated by arrowheads, visualized at ×20 magnification (scale bars: 200 μm). (**J**) Quantification of osteocalcin^+^ cells per bone surface area. (**K** and **L**) TRAP staining. (**K**) TRAP^+^ cells are marked with arrowheads at ×20 magnification (scale bars: 20 μm). (**L**) Quantification of TRAP^+^ cells per bone surface area. (**M**) Representation of eroded surface (ES/BS) in the plot. In **H** and **M**, *n* = 4 for each group. Sample sizes were *n* = 5 for other measurements. The results are presented as box-and-whisker plots. Statistical significance was assessed via 1-way ANOVA and Tukey-Kramer post hoc test. **P < 0.01, ***P < 0.001.

In further investigating the underlying mechanisms, our analysis revealed that Piezo1 expression in osteocytes was significantly reduced in the DEX-treated group, whereas the group treated concurrently with DEX and Yoda1 maintained expression levels comparable to the vehicle-treated group (Figure 3, A and B). In contrast, Sost expression in osteocytes was significantly upregulated in the DEX-treated group, but was attenuated by Yoda1 treatment (Figure 3, C and D). Silver staining demonstrated a reduction in the dendritic length of the LCN in the DEX-treated group, whereas the Yoda1-treated group showed no change compared to the vehicle-treated group (Figure 3, E and F). These findings were further supported by phalloidin staining (Figure 3, G and H). On the other hand, the osteocalcin-positive cell counts on trabecular bone surfaces significantly declined in the DEX-treated group, but were preserved by Yoda1 treatment (Figure 3, I and J). The number of TRAP-positive cells and the eroded surface (ES/BS) on the trabecular bone did not show significant differences between the DEX- and vehicle-treated groups. Notably, the concomitant administration of DEX and Yoda1 significantly reduced both the number of TRAP-positive cells and the eroded-surface measurements (Figure 3, K–M).

### Yoda1 attenuates the diminished response to mechanical stress induced by glucocorticoids.

**Figure 4:**
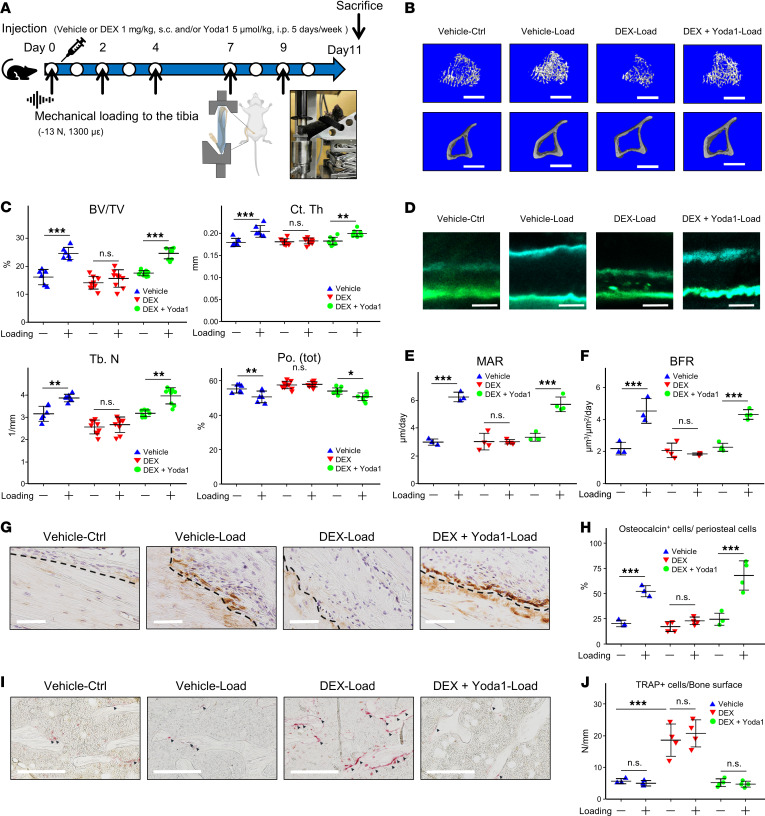
Yoda1 reverses the glucocorticoid-induced attenuation of mechanically driven bone responses. (**A**) Protocol for tibial mechanical loading and DEX and Yoda1 administration schedule: mice received DEX (1 mg/kg s.c.), Yoda1 (5 μmol/kg i.p.), or vehicle (distilled water s.c. and 5% ethanol i.p.) in 5 injections over 1 week. The left tibiae were subjected to axial mechanical loading 2–3 times weekly using an ElectroForce 5500 system. Each loading session consisted of 40 cycles of a trapezoidal waveform applying −13 N for 0.1 seconds, with 10-second intervals. Experimental groups consisted of Vehicle, DEX, and DEX+Yoda1. (**B**) Microcomputed tomography scans of both cortical and trabecular bone sections (scale bar: 250 μm). (**C**) Microcomputed tomography evaluations, including BV/TV, Ct. Th., Tb. N., and Po. Tot (*n* = 6 for Vehicle, *n* = 9 for DEX, and *n* = 9 for DEX+Yoda1). (**D**) Images of tetracycline and calcein labeling at ×100 magnification (scale bars: 5 μm). Labeling durations were 2 days for tetracycline and 1 day for calcein. (**E**) MAR. (**F**) BFR. For panels **E** and **F**, *n* = 3 for each group. (**G** and **H**) IHC for osteocalcin expression. (**G**) Periosteal bone surfaces, demarcated by a dashed line (original magnification, ×20; scale bars: 200 μm). (**H**) The ratio of osteocalcin^+^ cells on the periosteal surface. (**I** and **J**) TRAP staining. (**I**) TRAP^+^ cells were identified with arrowheads at ×20 magnification (scale bars: 20 μm). (**J**) Quantification of TRAP^+^ cells per bone surface area. For panels **H** and **J**, *n* = 3–4 for each group. All data are expressed as the mean ± SD. Statistical significance was determined using a 1-way ANOVA followed by a Tukey-Kramer post hoc test. *P < 0.05; **P < 0.01; ***P < 0.001. NS, not significant.

In vivo, axial tibial loading was performed for 11 days in mice to directly assess the compromised mechanical stress response in the context of GIOP (Figure 4A). However, compared with the vehicle-treated group, the DEX-treated group did not respond to mechanical loading. In contrast, the group treated with concurrent DEX and Yoda1 administration exhibited a significant enhancement in BV/TV, Tb. N, and Ct. Th. in response to the loading intervention, similar to the vehicle-treated group (Figure 4, B and C, and Supplemental Figure 5). Similarly, the Po. tot was significantly reduced by mechanical loading in both the vehicle- and combined DEX- and Yoda1-treated groups, whereas the DEX-treated group lacked this loading response (Figure 4C). Yoda1-only treatment did not significantly increase bone mass beyond normal mechanical loading (Supplemental Figure 6). Furthermore, the MAR and BFR were significantly elevated by mechanical loading in the vehicle- and combined DEX- and Yoda1-treated groups, but the DEX-treated group did not exhibit a response (Figure 4, D–F). Osteocalcin-positive periosteal cells were significantly increased by loading in both the vehicle- and DEX- and Yoda1-treated groups, but the DEX-treated group did not exhibit a response (Figure 4, G and H). In contrast, the number of TRAP-positive cells on the trabecular bone surface was significantly higher in the DEX-treated group, regardless of mechanical loading, whereas this was not observed in the vehicle- or DEX- and Yoda1-treated groups (Figure 4, I and J). Additionally, the effect of DEX and Yoda1 on the differentiation of bone marrow–derived macrophages (BMDMs) into osteoclasts was investigated, revealing that Yoda1 partially suppressed osteoclast differentiation (Supplemental Figure 7).

### Yoda1 mediates resistance to DEX-induced changes in gene expression, osteocyte morphology, and Akt phosphorylation via calmodulin kinase II–dependent Ca^2+^ influx.

**Figure 5:**
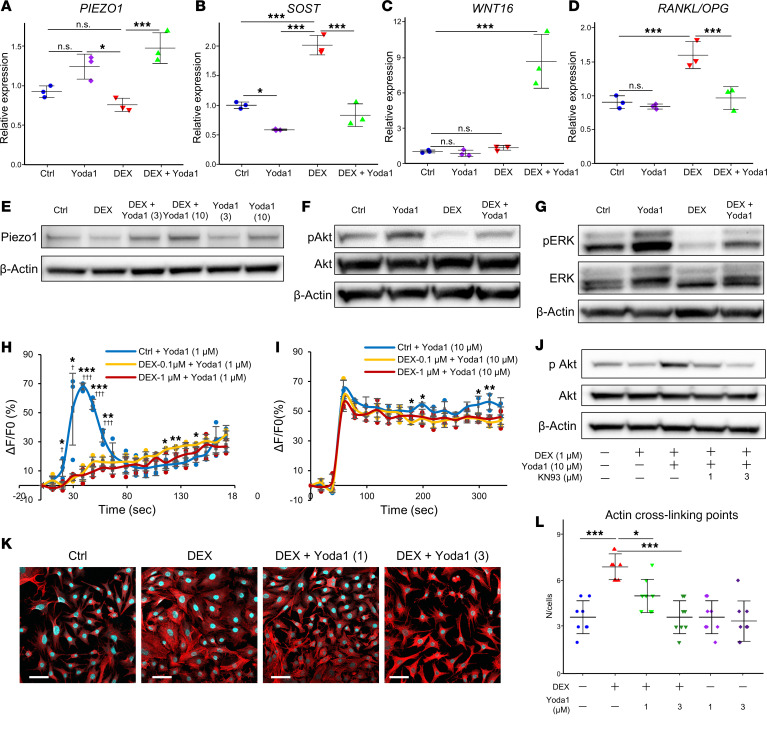
Effects of DEX and Yoda1 on human cortical bone and MLO-Y4 cells. (**A**–**D**) Human femoral neck cortical bone samples obtained from hip arthroplasty procedures were cleared of soft tissues. Subsequently, they were incubated overnight, followed by DEX and Yoda1 treatment for 6 hours (*n* = 3). Four experimental groups were established: Control (untreated), Yoda1 alone (10 μM), DEX alone (1 μM), and DEX+Yoda1 (combination of 1 μM DEX and 10 μM Yoda1). (**E**) WB analysis of Piezo1 and β-actin protein levels in MLO-Y4 cells following overnight treatment with DEX and subsequent 24-hour exposure to Yoda1. (**F** and **G**) WB analysis of Akt and ERK phosphorylation in response to DEX treatment and subsequent 2 hours of Yoda1 administration. (**H**) MLO-Y4 cells were incubated with DEX (0.1 and 1 μM) for 24 hours. Subsequently, all groups were treated with Yoda1 (1 μM) to monitor changes in Ca^2+^ influx (*n* = 3). (**I**) Yoda1 (10 μM) to monitor changes in Ca^2+^ influx (*n* = 3). (**J**) Impact of KN-93, a CaMKII selective inhibitor, on Akt phosphorylation in MLO-Y4 cells treated with DEX, preincubated with KN-93 for 2 hours, and then exposed to Yoda1 for 1 hour. (**K** and **L**) Morphological changes in F-actin structure in MLO-Y4 cells subjected to DEX and Yoda1 for 72 hours were visualized using rhodamine-phalloidin (see Supplemental Methods) and Hoechst 33342 staining. Images were acquired using an In Cell Analyzer 6000 at ×40 magnification (scale bars: 50 μm). (**L**) Actin cross-linking points were quantified (*n* = 8). Results are presented as the mean ± SD. Statistical significance was evaluated using a 1-way ANOVA followed by a Tukey-Kramer post hoc test. *P < 0.05, **P < 0.01, ***P < 0.001 for Control vs. DEX (0.1 μM) + Yoda1; †*P* < 0.05, ††*P* < 0.01, †††*P* < 0.001 for Control vs. DEX (1 μM) + Yoda1.

Upon examining a series of in vivo data suggesting a significant impact of osteocytes, human cortical bone organ culture assays were conducted to clarify gene expression effects for the pivotal role of bone metabolism (Figure 5, A–D). The *PIEZO1* and *PTGS2* expression levels were found to be significantly suppressed by DEX treatment, while Yoda1 preserved gene expression levels comparable to the control (Figure 5A and Supplemental Figure 8). Conversely, *SOST* and the ratio of *RANKL* to *OPG* expression were upregulated following DEX treatment, but remained at control levels after Yoda1 treatment (Figure 5, B and D). In addition, *WNT16* expression was markedly increased when cultures were treated with both DEX and Yoda1 (Figure 5C). At the protein level, Piezo1 expression in the MLO-Y4 cell line was reduced by DEX, but it was dose-responsively rescued by Yoda1 treatment (Figure 5E). In addition, the downstream phosphorylation signaling pathways in Piezo1 were investigated (Figure 5, F and G), revealing that Yoda1 treatment upregulated Akt and ERK phosphorylation, which were downregulated by DEX treatment. Moreover, Yoda1 could promote Akt and ERK phosphorylation that had been suppressed by DEX (Figure 5, F and G). Calcium influx in MLO-Y4 cells was quantitatively assessed to investigate the modulatory effects of DEX and Yoda1. Using Fluo-8 (see Supplemental Methods), a calcium indicator, Yoda1 was found to significantly accelerate Ca^2+^ influx. However, preincubation with 3 μM DEX attenuated the Yoda1-induced increase in Ca^2+^ influx, bringing it to levels comparable to Piezo1 knockdown achieved by siRNA interference (Supplemental Figure 9). In contrast, cotreatment with 1 μM DEX and a higher concentration of Yoda1 (10 μM) restored the Ca^2+^ influx to control levels (Figure 5, H and I). Further investigation was conducted to explore the downstream effects of Ca^2+^ on the phosphorylation of calcium/calmodulin-dependent protein kinase II (CaMKII), indicating that Yoda1 augments CaMKII phosphorylation (Supplemental Figure 10). Intriguingly, CaMKII inhibition by the selective inhibitor KN-93 (see Supplemental Methods) resulted in a dose-dependent decrease in Akt phosphorylation (Figure 5J). Morphological analysis of osteocytes revealed that actin cross-linking points increased following DEX treatment. However, this increase was dose-dependently attenuated by concurrent treatment with DEX and Yoda1 (Figure 5, K and L).

### Differential gene expression upon mechanical stress under DEX conditions and identification of Piezo1 transcriptional regulators.

**Figure 6:**
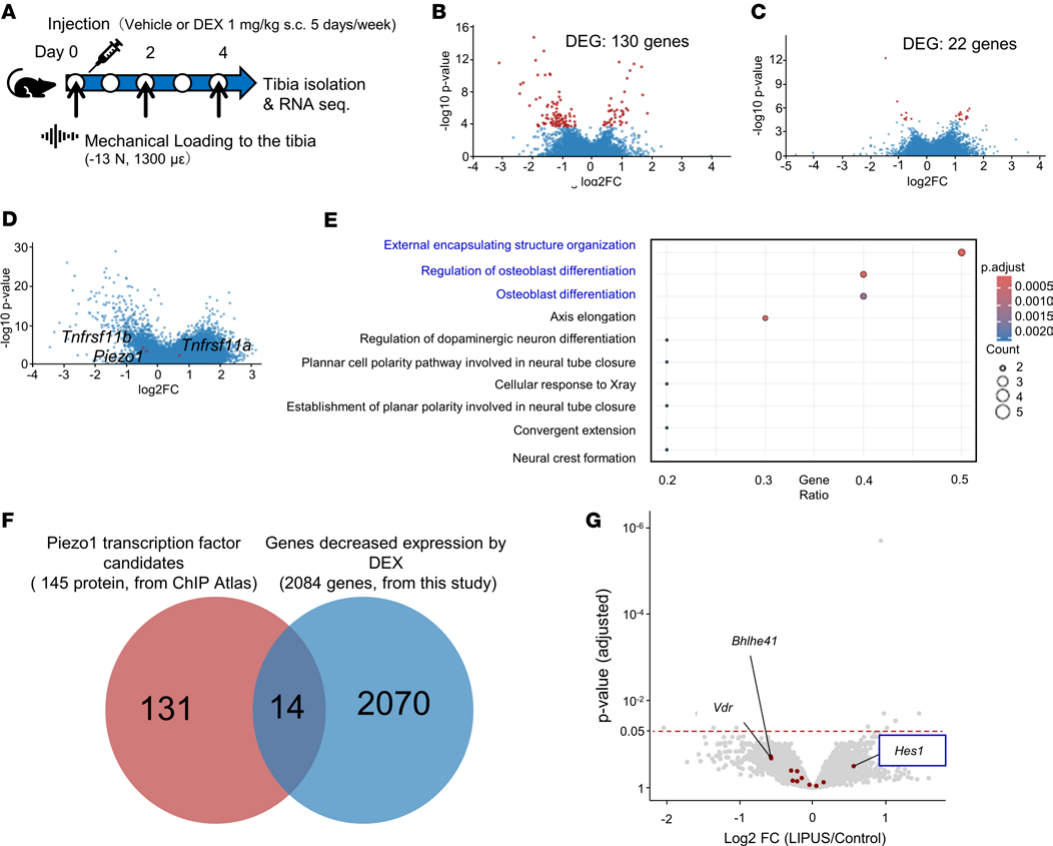
Differential gene expression analysis in response to mechanical stress under DEX treatment and investigation of the Piezo1 transcription factor. (**A**) Mouse tibial gene expression profiling was performed following mechanical loading using RNA-seq. Subcutaneous injections of 1 mg/kg DEX (DEX group) or distilled water (Vehicle group) were administered 5 times over 7 days. The left tibia underwent mechanical stress (40 cycles of −13 N force for 0.1 seconds at 10-second intervals, 3 times weekly) using an ElectroForce 5500 system. The right tibia remained unloaded as a control. Tibiae were flash-frozen for RNA extraction and sequencing 4 hours after final loading on day 5. (**B** and **C**) Differentially expressed genes (DEGs) are presented for the vehicle (**B**) and DEX (**C**) groups upon loading. Statistical significance was determined using the DESeq2 Wald test, highlighted with red dots for adjusted *P* values of less than 0.05. (**D**) Comparisons between vehicle- and DEX-treated groups under loading conditions revealed changes in genes such as *Piezo1* (log_2_ fold change [log_2_FC] = −0.38, adjusted *P* value [*P*_adj_] = 3.0 × 10^–3^), *Tnfsf11b* (log_2_FC = −0.48, *P*_adj_ = 7.0 × 10^–4^), and *Tnfrsf11a* (log_2_FC = 0.67, *P*_adj_ = 1.8 × 10^–2^). (**E**) Gene Ontology (GO) overrepresentation analysis revealed impaired response to mechanical stress under DEX, with terms ranked by gene ratio and the bone-related terms highlighted (adjusted for multiple comparisons using an FDR). (**F**) A Venn diagram integrates data on Piezo1 transcription factor candidates from the ChIP atlas and RNA-seq. (**G**) Gene expression changes upon mechanical stress (low-intensity pulsed ultrasound, LIPUS) in MLO-Y4 cells are depicted. Piezo1 transcription factor candidates (mapped from previous research by Shimizu et al., ref. 19; GEO GSE162674) are highlighted in red. *Hes1*, hairy and enhancer of split 1; *Vdr*, vitamin D receptor; *Bhlhe41*, basic helix-loop-helix family, member 41.

To investigate the mechanisms of glucocorticoid-mediated mechanical stress response, an RNA-seq analysis was conducted comparing the mouse tibial axial loading responses between the vehicle- and DEX-treated groups over 5 days (Figure 6A). To ensure data quality, principal component analysis and hierarchical clustering were employed. The DEX-4 dataset was identified as an outlier and consequently removed from the study (Supplemental Figure 11). The analysis revealed 130 differentially expressed genes (DEGs) associated with mechanical loading in the vehicle-treated group (Figure 6B). In contrast, the DEX-treated group exhibited only 22 DEGs following loading (Figure 6C). Comparative gene expression analysis between the vehicle- and DEX-treated groups during loading periods showed that *Piezo1* and *Tnfrsf11b* (OPG) expression significantly decreased, whereas *Tnfrsf11a* (RANK) expression increased in the DEX-treated group (Figure 6D). Gene Ontology (GO) overrepresentation analysis, specifically focusing on the altered response to mechanical stress under DEX treatment, showed significant enrichment for bone-related terms, including organization of external encapsulating structures, regulation of osteoblast differentiation, and osteoblast differentiation (Figure 6E). To identify transcription factors that may affect Piezo1, 145 genes were selected as candidates from the ChIP Atlas (https://chip-atlas.org/ Accessed April 20, 2022 and February 28, 2023.), an open-source database (Supplemental Figure 12). By integrating these Piezo1 transcription factor candidates with RNA-seq data using a Venn diagram, 14 genes were identified (Figure 6F). To further narrow down the focus to osteocyte-specific genes responsive to mechanical stimuli, these 14 candidate genes were mapped to a previously reported dataset (NCBI Gene Expression Omnibus [GEO] accession GSE162674). In this dataset, MLO-Y4 cells were treated with low-intensity pulsed ultrasound (LIPUS) as a mechanical stressor (19). This analysis revealed that *Hes1* was the only gene that showed upregulation following LIPUS treatment (Figure 6G).

### Hes1 as a regulatory transcription factor for Piezo1 modulation by DEX and Yoda1.

**Figure 7:**
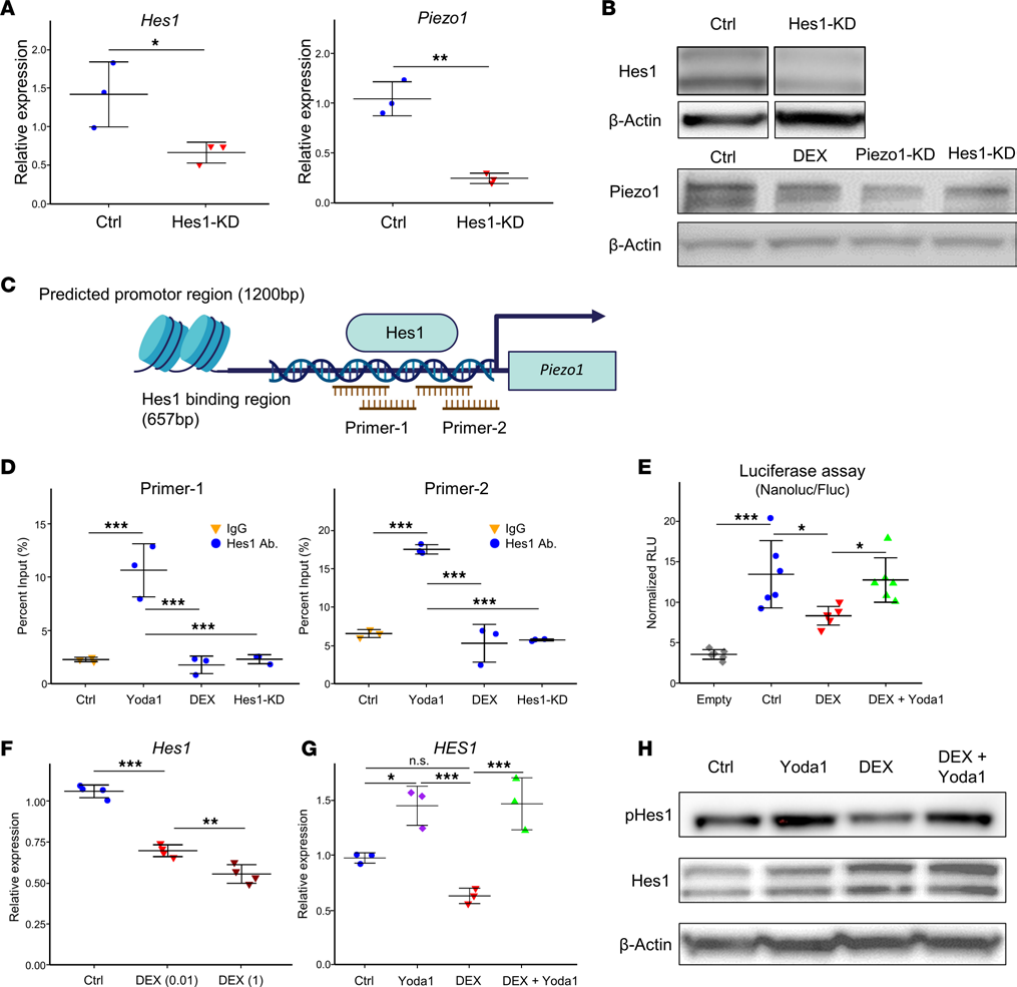
Hes1 is a regulatory transcription factor of Piezo1 modulated by DEX and Yoda1. (**A**) The impact of *Hes1* knockdown on Piezo1 expression, as assessed by qPCR following *Hes1* siRNA or control RNA electroporation in MLO-Y4 cells, 2 days after electroporation. Results are presented as mean ± SD (*n* = 3) and were analyzed using a 2-tailed Student’s *t* test with a 95% confidence interval. *P < 0.05, **P < 0.01. (**B**) WB analysis for Hes1 and Piezo1 was conducted after siRNA transfection and overnight incubation with DEX (1 μM). (**C**) A schematic of the *Piezo1* promoter (1200 bp) with the Hes1 binding site (657 bp) and highlighted CUT&RUN assay primers. (**D**) CUT&RUN assay results after siRNA transfection and overnight incubation, followed by treatment with DEX (1 μM) or Yoda1 (10 μM) for 1 day. The Ctrl group utilized rabbit IgG for immunoprecipitation, while other groups used an anti-Hes1 antibody (*n* = 3). (**E**) Luciferase assay after vector electroporation and overnight incubation, followed by DEX (1 μM) overnight and subsequently Yoda1 (10 μM) treatment for 4 hours. Constructs included Empty (empty pNL3.1 vector) and Ctrl (pNL3.1 with Hes1 binding region), *n* = 6. (**F**) The DEX dose-response effect on *Hes1* expression in MLO-Y4 cells was analyzed using qPCR 4 hours after treatment (*n* = 4). (**G**) The effects of DEX (1 μM) and Yoda1 (10 μM) on *HES1* mRNA levels in human cortical bone organ cultures were measured by qPCR 6 hours after treatment (*n* = 3). (**H**) Analysis of Hes1 phosphorylation upon treatment with DEX (1 μM), followed by Yoda1 (10 μM) for 4 hours. Data are presented as mean ± SD. Results were analyzed with a 1-way ANOVA and the Tukey-Kramer post hoc test. *P < 0.05; **P < 0.01; ***P < 0.001. NS, not significant.

In this study, we investigated whether Hes1 acts as a transcription factor for Piezo1 (Figure 7). Upon *Hes1* knockdown in MLO-Y4 cells, Piezo1 expression was found to be reduced at both the mRNA and protein levels (Figure 7, A and B). To further confirm the binding of Hes1 to the *Piezo1* gene, cleavage under targets and release using nuclease (CUT&RUN) assays were performed using 2 sets of primers (Supplemental Table 4) designed to target the Hes1 binding region (Supplemental Figure 9), as identified in the ChIP Atlas database (Figure 7, C and D). Treatment with Yoda1 significantly enhanced the amplification of the Hes1 binding region in MLO-Y4 cells, whereas DEX treatment and *Hes1* knockdown did not (Figure 7D). Furthermore, a luciferase assay revealed that *Piezo1* transcriptional activity was inhibited by DEX treatment and significantly rescued by Yoda1 (Figure 7E). Furthermore, the impact of DEX and Yoda1 treatment on Hes1 expression at the gene and protein levels was also investigated (Figure 7, F–H). DEX treatment was found to induce a dose-dependent decrease in *Hes1* mRNA levels, while Yoda1 treatment markedly increased *Hes1* mRNA levels (Figure 7, F and G). Notably, Hes1 phosphorylation was found to decrease with DEX treatment, while increasing with Yoda1 treatment (Figure 7H). In general, Hes1 expression is regulated by Notch signaling, the canonical pathway for Notch1 activation as evidenced by the observation of cleaved Notch1 (20). Yoda1 treatment increased cleaved Notch1 levels (Supplemental Figure 14).

### Integrated analysis of cortical bone response to mechanical stress in GIOP: insights from patient data and mouse tibia RNA-seq.

**Figure 8:**
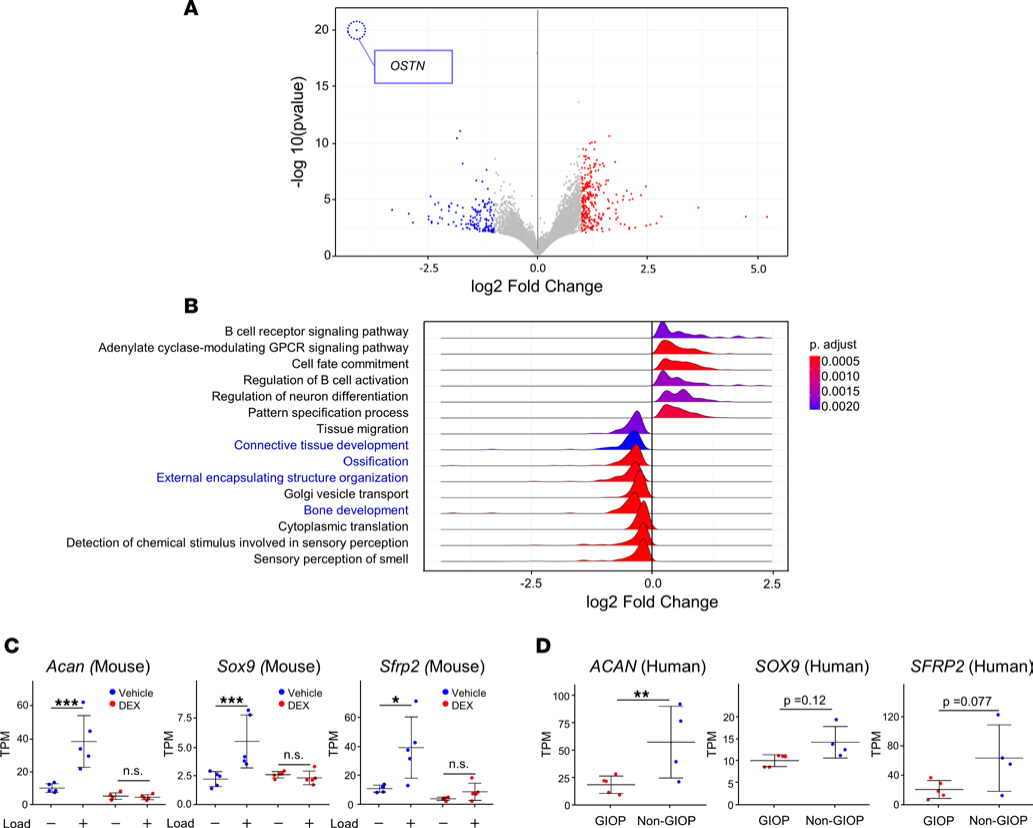
An integrated analysis of the attenuated mechanical stress response in GIOP, based on cortical bone data from both patients with GIOP and mouse tibial loading RNA-seq. (**A** and **B**) Relative gene expression analysis between patients with and without GIOP was conducted using Wald’s test implemented in DESeq2. (**A**) Markedly altered gene expression in GIOP is depicted by blue (decrease) and red (increase) spots (*P* < 0.05). For instance, *OSTN* (osteocrin) log_2_ fold change is −4.13 (*P*_adj_ = 2.3 × 10^–16^). (**B**) Gene set enrichment analysis based on the log_2_ fold change of genes between patients with and without GIOP, with bone-related terms highlighted. (**C** and **D**) Gene expression changes in the mouse tibia (featuring *ACAN*, *SOX9*, and *SFRP2*) and the human femoral neck. Data are expressed as mean ± SD (*n* = 4–5 per group). Statistical analysis was performed using Wald’s test according to the DESeq2 workflow and the appropriate statistical model. *P < 0.05; **P < 0.01; ***P < 0.001, with FDR correction applied to account for multiple error risks. NS, not significant.

We observed an altered response to mechanical stress during DEX treatment and aimed to identify genes common to both the GIOP mouse model and patients with GIOP (Figure 8). RNA-seq analysis was performed on RNA extracted from the cortical bone, including the periosteum, of age-matched female patients with GIOP and non-GIOP counterparts (Table 1 and Supplemental Table 1). This analysis identified osteocrin (*OSTN*), which is abundantly expressed in the periosteum and has been shown to promote osteoblast differentiation in response to mechanical loading (21), as the most profoundly downregulated gene in the GIOP group (Figure 8A). GO analysis revealed significant suppression of bone-related terms in the cortical bone of patients with GIOP, including bone development, organization of external encapsulating structures, ossification, and connective tissue development (Figure 8B). To identify genes that are translationally affected by glucocorticoid treatment and responsive to mechanical stress, RNA-seq data from both mouse models and patients with GIOP were integrated, leading to the identification of 10 candidate genes (Table 2 and Table 3). In the mouse model, several genes, including *Acan* (aggrecan), *Sox9* (SRY-related HMG box gene 9), and *Sfrp2* (secreted Frizzled-related protein 2)*,* were significantly upregulated in response to mechanical stress. However, this upregulation was blunted by DEX treatment (Figure 8C and Supplemental Figure 15A). Moreover, these genes displayed a tendency for higher expression levels in non-GIOP patients than in patients with GIOP (Figure 8D, Supplemental Figure 15B).

### DEX and Yoda1 influence Piezo1 expression and osteoblast differentiation in human PDCs.

**Figure 9:**
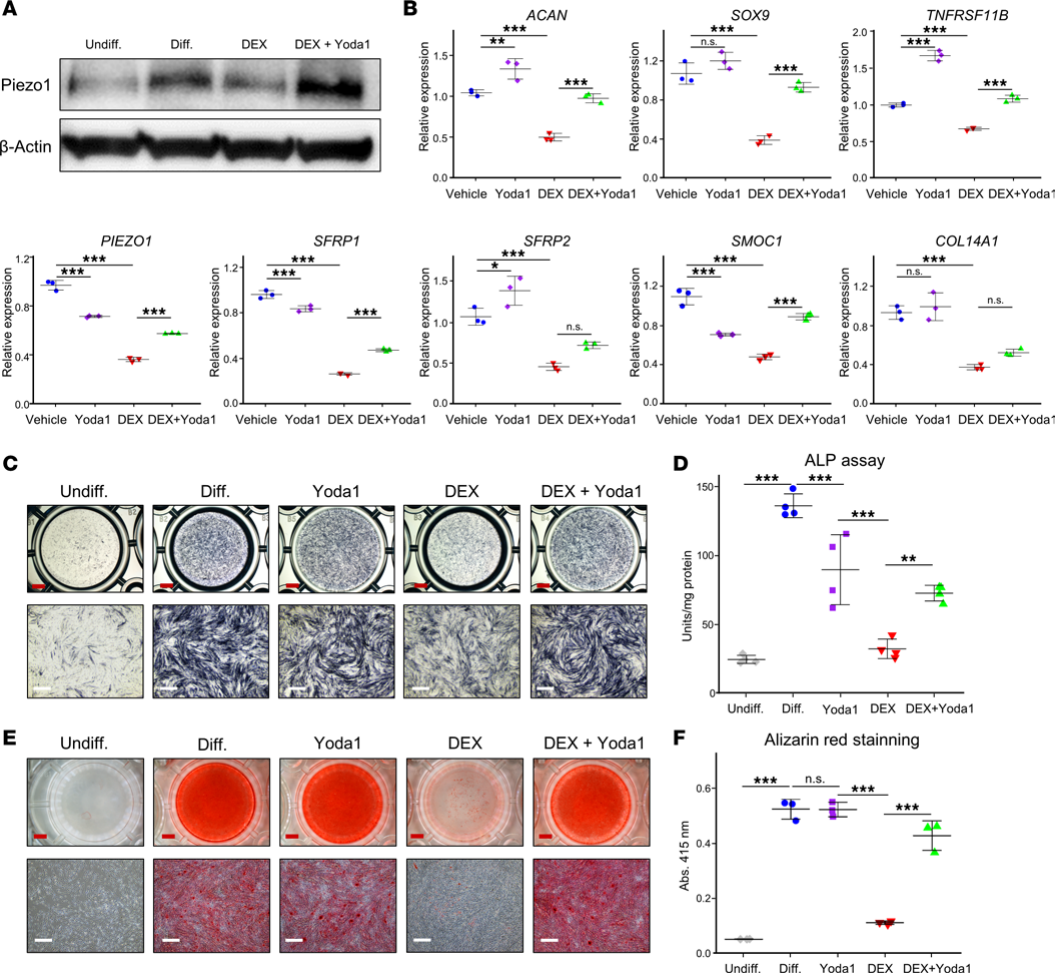
Impact of DEX and Yoda1 on Piezo1 expression and osteoblast differentiation in PDCs. (**A**) Piezo1 expression evaluation during PDC osteogenesis. PDCs isolated from the femur during knee arthroplasties were treated with collagenase type II overnight and differentiated using the STEM PRO Osteogenesis Kit (see Supplemental Methods). After 3 days, the media were replaced, and cells were treated with DEX (1 μM) overnight, followed by a 4-hour exposure to Yoda1 (10 μM) before protein extraction for WB. Five experimental groups were established: Undiff. (undifferentiated PDCs), Diff. (differentiated PDCs), Yoda1 (Yoda1-treated after differentiation), DEX (DEX-treated after differentiation), and DEX+Yoda1 (DEX and subsequent Yoda1 after differentiation). (**B**) PDC gene expression was assessed through qPCR following overnight DEX (1 μM) and subsequent 4-hour exposure to Yoda1 (10 μM) (*n* = 3). (**C** and **D**) Osteoblast differentiation was initiated in PDCs with DEX (1 μM) and Yoda1 (1 μM) addition 1 day after induction. (**C**) Alkaline phosphatase (ALP) staining and (**D**) ALP assay were conducted on day 14 (*n* = 4). (**E** and **F**) Alizarin red S staining was performed on day 21 after differentiation to evaluate mineralization at low (×1.2) and high (×4) magnifications. Scale bars: 2 mm (low) and 500 μm (high). (**F**) Quantification of mineralization through solubilization in 5% formic acid and measuring absorbance at 415 nm (*n* = 3). Data are presented as mean ± SD. A 1-way ANOVA coupled with a Tukey-Kramer post hoc test was employed for statistical analysis. *P < 0.05; **P < 0.01; ***P < 0.001. NS, not significant.

Translational analysis of cortical bone, including the periosteum, in both a mouse model (tibia) and a patient with GIOP in the femoral neck revealed a list of 10 genes that are related to osteoblast and chondrocyte functions. This finding suggests that there is an inhibition in the differentiation of preosteoblasts to osteoblasts in GIOP. To further investigate this, Piezo1 expression during osteoblast differentiation was compared in human PDCs obtained from total knee arthroplasty and MC3T3-E1 cells (Figure 9A and Supplemental Figure 16). Human PDCs showed an upregulation of Piezo1 expression during the early stage of osteoblastic differentiation, which was suppressed by DEX treatment. However, cotreatment with DEX and Yoda1 resulted in a marked induction of Piezo1 expression (Figure 9A). Conversely, MC3T3-E1 cells did not exhibit any notable changes in Piezo1 expression (Supplemental Figure 11). Focusing on PDCs, a quantitative PCR (qPCR) analysis was performed on the list of 10 genes, and 6 genes (*ACAN*, *SOX9*, *SFRP1*, *SFRP2*, *SMOC1* [SPARC-related modular calcium binding 1], and *COL14A1* [collagen type XIV α1 chain]) were found to be significantly downregulated by DEX treatment. However, this effect was significantly reversed by Yoda1 treatment (Figure 9B). To further assess the impact of DEX and Yoda1 treatment on PDC osteoblast differentiation, the outcomes of alkaline phosphatase (ALP) staining, ALP activity assays, and alizarin red S staining (Figure 9, C–F) were assessed. DEX treatment notably reduced ALP activity, but this effect was mitigated when DEX and Yoda1 treatment were used concurrently (Figure 9D). Similarly, alizarin red S staining, which was significantly reduced by DEX treatment, showed less suppression when DEX and Yoda1 were combined (Figure 9F). Additionally, DEX treatment was found to slightly reduce the number of live cells, but this was not affected by Yoda1 treatment (Supplemental Figure 17).

## Discussion

**Figure 10:**
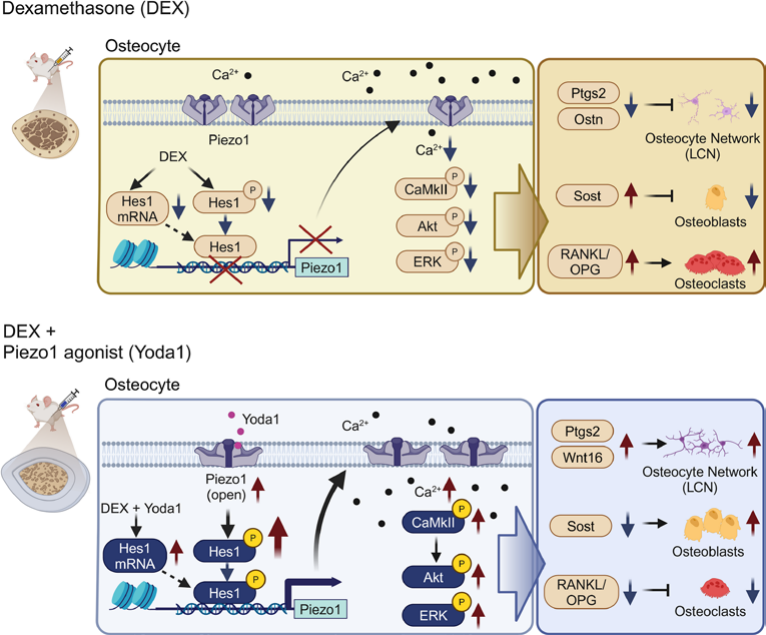
Yoda1-induced enhancement of osteocyte function via Hes1/Piezo1 signaling. Yoda1 enhances Piezo1 expression through Hes1 activation, increasing CaMKII and Akt phosphorylation in osteocytes. This results in an improved lacuno-canaliculi network (LCN), reduced sclerostin production, and a balanced RANKL/OPG ratio, effects that are diminished by DEX.

In this study, we demonstrated a reduction of Piezo1 expression in the osteocytes of patients with GIOP and a mouse model of GIOP. Notably, concurrent treatment with DEX and Yoda1 prevented disease progression in the mouse model. We conducted studies using GIOP prevention models. Unlike primary osteoporosis, where treatment is typically initiated after the disease has progressed, GIOP allows for therapeutic intervention from the start of glucocorticoid administration. Therefore, we believe that research on preventive models of GIOP is quite important. Bergström et al. reported a lack of response to axial tibial loading in a GIOP mouse model (22). However, there have been no reports on the protective effects of Yoda1 on GIOP progression. Interestingly, our findings revealed that the combination of DEX and Yoda1 treatment effectively prevented bone strength loss and bone mass reduction in both cortical and trabecular bone. The effect was particularly pronounced in cortical bone, as evidenced by increased width and reduced porosity (Figures 2 and 3). It is well known that glucocorticoids induce a rapid increase in bone resorption in the early stages, followed by a medium- to long-term decrease in bone formation (23). In Figure 3L, the DEX administration period was 1 month, and TRAP-positive cells/BS did not change significantly. In contrast, in Figure 4J, the DEX treatment duration was only 11 days, and TRAP-positive cells/BS were significantly increased. This difference in the effects on bone resorption may be attributed to the difference in evaluation periods. The principal pharmacological activities of Yoda1 include stimulating the gene expression of *Piezo1*, which activates osteocyte LCN and promotes bone formation. Yoda1 also modulates *Sost* gene expression and reduces the count of osteoclasts and the extent of eroded surfaces in vivo (Figures 3 and 4). Nevertheless, the impact of Yoda1 on osteoclast differentiation from BMDMs was only partial (Supplemental Figure 3). Consistent with these findings, Li et al. reported that Yoda1 inhibits osteoclast differentiation in vitro in cocultures of osteocytes and BMDMs (18), suggesting that osteocytes mediate this inhibitory effect. Importantly, Yoda1 treatment during human cortical bone organ culture experiments reduced the RANKL/OPG ratio, which is critical for osteoclast differentiation (Figure 5). The in vivo data series supports the notion that Yoda1 enhances bone formation while simultaneously reducing bone resorption. This study provides insights into the mechanism of the Piezo1/Hes1 pathway within the osteocytes of GIOP (Figure 10).

Several studies have shown that DEX affects the expression of various genes (24, 25), but there have been no reports suggesting that glucocorticoids downregulate Piezo1 expression. Hendrick et al. observed an upregulation of Piezo1 in cartilage cells mediated by Yoda1 (26), but reports on osteocytes are lacking. Sasaki et al. demonstrated that Yoda1 accelerated Akt phosphorylation in MLO-Y4 cells (27). In our findings, DEX suppressed Akt and ERK phosphorylation, which was reversed by Yoda1, providing insights as these effects have not been previously described in osteocytes to the best of our knowledge. This phosphorylation cascade, which results in the inhibition of glycogen synthase kinase 3 (GSK3β) and subsequent suppression of β-catenin degradation, is a critical component of the Wnt canonical pathway (28). Downregulation of Wnt signaling has been frequently reported in the context of GIOP (29). Our study contributes to this discussion by demonstrating Yoda1-mediated downregulation of Sost and the RANKL/OPG ratio (Figure 5). Sost acts as a potent inhibitor of Wnt signaling, and, according to Sato et al., an anti-Sost antibody reduces symptoms in a GIOP mouse model by downregulating RANKL/OPG (30). Furthermore, Miyazaki et al. reported that both Yoda1 and mechanical stress induce Wnt16 expression in mouse odontogenic cells (31), but such effects have not been studied in the context of human cortical bone organ cultures. In addition, our data revealed that the enhancement of Akt phosphorylation by Yoda1 can be inhibited by preincubation with KN-93, a known CaMKII inhibitor. A previous study reported that Piezo1 deletion leads to diminished phosphorylation of CaMKII (14), but currently, there are no reports indicating that phosphorylation of CaMKII acts upstream of Akt phosphorylation in osteocytes. Gao et al. highlighted that DEX alters the morphology of MLO-Y4 cells (32). Our study builds on this by examining actin cross-linking points using an In Cell Analyzer 6000, suggesting their potential relevance to cellular adhesion (33). However, further confirmation through more detailed experimentation is required to support this inference.

In this study, RNA-seq analysis of mouse axial tibial loading, supplemented with data from open sources such as the ChIP Atlas and prior RNA-seq datasets, identified Hes1 as a potential transcription factor for Piezo1. Although there is currently no direct evidence of Hes1 regulating Piezo1 transcription, Caolo et al. have shown that Piezo1 is involved in the force sensitivity of a disintegrin and metalloproteinase domain-containing protein 10 (ADAM10) and neurogenic locus Notch homolog protein 1 (Notch1), subsequently activating Notch1 target genes, including *Hes1* (20). The study also found that Yoda1 upregulated *HES1* mRNA expression in human cortical bone organ cultures and enhanced Hes1 phosphorylation (Figure 7). Sigita et al. suggested that phosphorylated Hes1 functions as an active transcription factor (34), implying its pivotal role in the transcriptional regulation of Piezo1. Nonetheless, further investigations are required to confirm this hypothesis. In addition, Revello et al. noted an overlap between Hes1 target genes and numerous glucocorticoid-responsive genes, suggesting a reciprocal relationship in their expression patterns (35). Recent findings have also highlighted the importance of Hes1 in the differentiation of osteoprogenitor cells into osteoblasts (36). Overall, understanding Hes1 function holds promise for advancements in the field of bone metabolism beyond GIOP.

In this study, RNA-seq analysis was performed on cortical bone samples from patients with and without GIOP and revealed a marked downregulation of *OSTN* in patients with GIOP (Figure 8A). Wang et al. have demonstrated that *OSTN* expression is regulated by the transcription factor SP7, and *OSTN* overexpression can enhance dendritic formation in osteocytes with *Sp7* knockout (37). Additionally, *Ostn* overexpression in a GIOP mouse model has been shown to partially reverse the glucocorticoid-induced reduction in osteocyte dendritic length (38). To the best of our knowledge, there are no reports using actual bone tissue from patients with GIOP to confirm these findings. This assertion is further corroborated by our data showing a significant downregulation of LCN in cortical bone sections from patients with GIOP (Figure 1). Our study enhances our understanding of how DEX affects bone tissue at the molecular level under mechanical stress. We identified 10 genes that exhibited changes in their expression levels, as revealed by our translational RNA-seq analysis (Figure 8). *Acan* and *Sox9*, for instance, are primarily expressed in cartilage and periosteal osteoprogenitor cells and play crucial roles in bone tissue repair after fracture (39–41). These genes respond to mechanical stress by upregulating their expression in cartilage cells. Furthermore, *Sfrp1* and *Sfrp2*, recognized as Wnt pathway inhibitors that act by binding to various Wnt ligands, exhibit an interesting regulatory mechanism (42–44). Lau et al. found that *Sfrp2* is upregulated in response to mechanical loading in bone tissue, suggesting a negative feedback mechanism for mechanical stress–induced Wnt signaling (45). However, Castro et al. noted a complex function of *Sfrp2*, where its deletion in skeletal stem cells hindered bone healing (43), and its activity as a Wnt pathway agonist was concentration dependent (44), indicating an intricate regulatory system that is not yet fully understood. Takahata et al. identified SMOC1 as an essential element in the differentiation process from osteoprogenitor cells to osteoblasts, which is regulated by Runt-related transcription factor 2 (Runx2) (46). We propose that changes in the expression of these genes may influence the differentiation and recruitment of periosteal osteoprogenitors to the osteoid surface. Notably, our histomorphometric analysis revealed a significant reduction in the bone surface occupied by osteoblasts (Ob. S/OS) following DEX treatment, which was reversed with concurrent DEX and Yoda1 treatment (Figure 2E).

We demonstrated that the differentiation of human PDCs to osteoblasts was inhibited by DEX treatment, but this inhibition was mitigated by concurrent treatment with DEX and Yoda1. This suggests that, in addition to osteocytes, periosteal cells could be implicated in the progression of GIOP (Figure 9). Supporting this, our in vivo data are consistent with our in vitro findings, showing a decrease in the prevalence of osteocalcin-positive cells in the periosteum following DEX treatment. However, this decline was prevented by the combined administration of DEX and Yoda1 (Figure 3, I and J). To the best of our knowledge, no previous studies have investigated the reduced periosteal cell response to mechanical stress caused by DEX, both in vivo and in vitro. One limitation of this study is that it did not fully elucidate the complex interactions between osteocytes and periosteal cells. Nevertheless, considering that osteocytes can regulate the functions of other cells through the LCN (47), it is conceivable that an intricate network involving osteocytes and periosteal cells exists. This study focused on the impact of Yoda1 on osteocytes, but we were unable to analyze osteocyte-specific conditional knockout mice. This limitation should be addressed in future investigations. The ubiquitous role of Piezo1 across multiple organ systems and its targeted delivery to bone are crucial for maximizing therapeutic efficacy while minimizing the risks of adverse effects (12). In the context of GIOP treatment, the conjugation of bone-specific targeting peptides or bisphosphonates to Piezo1 agonists represents a strategic approach to overcome the challenge of achieving site-specific drug action (48).

In summary, our study revealed downregulated LCN and Piezo1 expression in cortical bone tissue from patients with GIOP. Furthermore, a GIOP mouse model demonstrated a diminished response to mechanical stress, highlighting the critical role of Piezo1 in disease progression. Administration of Yoda1, a Piezo1 activator, counteracted the impaired mechanical stress response in the GIOP model. This research not only provides insights into the pathophysiology of GIOP, but also offers potential therapeutic strategies for the disease by targeting Piezo1 activation and simulating mechanical stress.

## Methods

Further information can be found in Supplemental Methods.

### Sex as a biological variable.

Our study investigated female patients with and without GIOP, as well as male mice, and similar findings were reported. This is consistent with a recent study by Palmowski et al., who reported that the association of glucocorticoid use with reduced BMD in patients was not influenced by sex (49). Additionally, various studies have used GIOP models in both male and female mice (50). In our research, the individuals with and without GIOP were postmenopausal women, which raises concerns about the potential confounding effects of decreased estrogen levels. However, our study of the male GIOP mouse model revealed several similarities with female patients with GIOP, highlighting one of the key areas of investigation.

### Cell line culture.

MLO-Y4 cells (Karafest, CVCL_M098) were cultured in α-minimal essential medium (αMEM; Nacalai Tesque, 21444-05) supplemented with 5% fetal bovine serum (FBS; Hyclone, SH30396.03), 5% fetal calf serum (Gibco, 16010-159), and 1% antibiotic-antimycotic solution (Sigma-Aldrich, A5955). The cells were seeded onto collagen-coated 10-cm dishes (Iwaki, 4020-010). Similarly, MC3T3-E1 cells (Riken, RCB1126) were incubated in αMEM containing 10% FBS and 1% antibiotic-antimycotic solution and cultured on collagen-coated 10-cm dishes.

### Human femoral cortical bone organ culture.

Cortical bone tissues from the femoral neck were collected from patients undergoing total hip arthroplasty and processed within 24 hours after surgery. Soft tissues, periosteum, and trabecular bone were carefully removed to isolate the cortical bone. The prepared cortical bone was then sectioned into fragments measuring approximately 1–2 cm. These fragments were placed in collagen-coated 10-cm dishes and incubated overnight at 37°C in αMEM supplemented with 10% FBS. Subsequently, the bone fragments were transferred to collagen-coated 6-well plates and treated concomitantly with DEX (1 μM) and Yoda1 (10 μM) for 6 hours. Following treatment, the samples were snap-frozen in liquid nitrogen and stored at −80°C for further analysis.

### Human PDC isolation and culture.

PDCs were harvested from the periosteum collected from the anterior side of the distal femur of patients undergoing total knee arthroplasty on the day of surgery. The harvested periosteum was carefully stripped of any adhering soft tissue and cut into approximately 1 cm^2^ squares. These periosteal explants were then rinsed with PBS and subjected to enzymatic digestion using a solution of Dulbecco’s modified Eagle medium (DMEM)/F-12 supplemented with GlutaMAX (Gibco, 10565018), 10% FBS, and 0.2% collagenase type II (Worthington, LS004176) at 37°C overnight (51). Following digestion, the tissue was strained through a 70-μm cell strainer and centrifuged for 3 minutes at 500*g*. The resulting cell pellet was plated onto 10-cm dishes preheated with collagen in DMEM/F-12 supplemented with GlutaMAX and 10% FBS. The cells were cultured until they reached confluence, detached using TrypLE Express (Gibco), and expanded up to passage 3. For cryopreservation, cells at passage 3 were stored in STEM-CELLBANKER (Zenoaq, CB045). PDCs up to passage 13 were used for subsequent experiments.

### RNA-seq analysis.

Total RNA was extracted from mouse tibiae and used for library construction using the SMARTer Stranded Total RNA-Seq Kit v2 – Pico Input Mammalian (Takara Bio, 634412). The resulting libraries were then sequenced on the HiSeq 3000 system (Illumina) following the manufacturer’s protocols.

### Data analysis for RNA-seq.

The raw RNA-seq data were obtained from the Research Institute for Microbial Diseases at Osaka University. Differential gene expression analysis was performed using the R software environment (version 4.3.1) and the RaNA-Seq pipeline (available at https://ranaseq.eu/home) (52). For data analysis, the DESeq2 package (53) coupled with Wald’s statistical test was used to determine differential gene expression. Statistical significance was determined using Wald’s test at a *P* value of less than 0.05. In cases where integrated analysis involved multiple RNA-seq datasets, FDR correction was applied to mitigate the risk associated with multiple comparisons.

### DEX and Yoda1 administration in animals.

Three-month-old male C57BL/6J mice were acquired from The Jackson Laboratory. DEX was prepared in sterile water for injection and administered s.c. at a dose of 1 mg/kg body weight. Yoda1 was initially dissolved in dimethyl sulfoxide to create a stock concentration of 40 mM. On the day of injection, Yoda1 was further diluted with 5% ethanol for i.p. administration at a dose of 5 μmol/kg body weight. Control animals received vehicle treatments: distilled water for injection (s.c.) and 5% ethanol (i.p.) for DEX and Yoda1. The experimental animals were divided into 3 groups: Vehicle, DEX, and DEX+Yoda1. Each group consisted of 10 animals and underwent a treatment regimen of 5 injections over 1 week, which was repeated weekly for a total of 4 weeks. For bone morphology analysis, on day 22, an alizarin complexone solution (Dojindo, 348-00093) in 2% sodium hydrogen carbonate was injected s.c. at a dose of 30 mg/kg body weight. Skeletons were labeled for 4 days. Subsequently, on day 27, calcein (Sigma-Aldrich, C0875) prepared in saline was administered s.c. at a dose of 10 mg/kg body weight, and labeling was conducted for 1 day. On day 29, under isoflurane anesthesia, the femurs were harvested for subsequent experimental analyses. This experiment was independently repeated twice for a 3-point bending test.

### In vivo axial tibial loading.

Three-month-old male C57BL/6J mice were used for axial tibial loading studies. The ElectroForce 5500 system (TA Instruments) was employed to apply mechanical loading to the left tibia. The loading protocol consisted of 40 cycles per session with a trapezoidal waveform, applying a force of −13 N (approximately 1300 με) for 0.1 seconds, followed by a 10-second rest interval, 2 to 3 times per week (54). Mice were anesthetized with isoflurane during each loading session. The right tibia of each mouse served as the internal, non-loaded control. The mice were divided into 3 treatment groups: Vehicle, DEX, and DEX+Yoda1. Each group comprised 10 mice, and the treatments were administered through 5 injections over 1 week. This cycle was repeated for a total duration of 2 weeks. For bone morphology analysis, tetracycline hydrochloride (Sigma-Aldrich, T-7660) was injected s.c. at a dose of 20 mg/kg body weight on day 6, and labeling was allowed on the skeleton for 2 days. On day 9, calcein (Sigma-Aldrich, C0875) prepared in normal saline was administered s.c. at a dose of 10 mg/kg body weight, and labeling was conducted for 1 day. On day 11, the mice were anesthetized with isoflurane, and their femurs were harvested for subsequent experimental analysis.

### Microcomputed tomography analysis.

The femurs and tibias were extracted from the 3-month-old mice after fixation in 70% ethanol and analyzed by microcomputed tomography using a Skyscan 1272 bench-top scanner (Bruker Corporation). Trabecular bone assessments were conducted in the metaphyseal regions of the tibiae or femur, specifically in the areas proximal to the growth plate at approximately 1 mm and extending to 500 μm. Cortical bone measurements were analyzed at the mid-diaphyseal sections, starting approximately 4 mm distal to the growth plates and continuing to 500 μm. The bones were scanned with an isotropic voxel size of 8 μm using an x-ray tube voltage of 90 kV and a current of 160 mA. The resulting scanned images were then interpreted and quantified using CTAn software (Bruker Corporation), where various structural parameters of the bone were evaluated.

### Three-point bending test and treatment groups.

The biomechanical integrity of the left femurs in mice was assessed using a 3-point bending test performed on an ElectroForce 5500. The mice were divided into 3 treatment groups: Vehicle, DEX, and DEX+Yoda1, with each group consisting of 10 mice. The treatments were administered via 5 injections over 1 week, and this regimen was repeated over 4 weeks. To simulate in vivo conditions, the bone samples were immersed in physiological saline before testing. The femurs were carefully placed on a supporting device (Square Medical), with the anterior surface facing upwards, ensuring that the 2 supporting contacts were 8 mm apart. The loading plunger, positioned perpendicularly to the longitudinal axis of the femurs, was centrally located above the bones. Initially, a preload of −0.5 N was applied to secure the bone in place. Then, a loading process was initiated at a uniform displacement rate of 0.1 mm/sec until a bone fracture occurred or a displacement of 2 mm was reached (55). Any unintended fractures resulting from malfunctions of the ElectroForce 5500 that led to uncontrollable displacement rates were documented as follows: 4 incidents in the vehicle group and 2 in the DEX+Yoda1 group. Throughout the testing process, load-displacement information was systematically recorded, allowing for the calculation of various structural properties, including maximum load (N), energy absorption (N•mm), and stiffness (N/mm), based on the acquired load-displacement curves.

### Human and mouse osteocyte morphology visualization using silver staining.

In brief, the deparaffinized sections of the human femoral neck and mouse femur were stained with a solution containing 33% silver nitrate, 0.7% gelatin (Nitta Gelatin, 211116), and 0.4% formic acid for 55 minutes in darkness at room temperature. The sections were then rinsed, treated with 5% sodium thiosulfate for 10 minutes, dehydrated, cleared, and mounted. Dendritic lengths within the stained lacunae and canaliculi were measured using ImageJ software (56).

### Osteocyte morphological assessment using phalloidin staining.

Mouse femurs were fixed in 4% paraformaldehyde in PBS for 24–48 hours, followed by decalcification in 5 M ethylenediamine tetraacetic acid, as previously reported (56). After fixation and decalcification, the metaphyseal regions were excised, and the marrow was extruded. Samples were then subjected to a series of sucrose solutions (10%, 15%, and 20%) for cryoprotection. Tissues were then embedded in OCT (optimal cutting temperature) compound following standard procedures. Cryosections of 50-μm thickness were prepared and placed onto 24-well slides. The cryosections were washed 3 times with PBS (1×) for 5 minutes each to remove all traces of OCT. An overnight incubation at 4°C with Blocking One Histo (Nacalai Tesque, 06349-64) was performed. Subsequently, the sections were stained with Alexa Fluor 488–conjugated phalloidin (Invitrogen, 12379) diluted 1:400 in PBS containing 5% Blocking One Histo and 1% Triton X-100 overnight at 4°C. After washing, nuclei were counterstained with 4′,6-diamidino-2-phenylindole (DAPI; DOJINDO, 340-07971), and slides were mounted using ProLong Diamond Antifade Mountant (Invitrogen, P36961).

### Bone histomorphometry.

Excised tibial and femoral bones were fixed in 70% ethanol for 3 days and then dehydrated in 30% sucrose. Following the Osteoresin Embedding Kit protocol (Fujifilm, 297-56001), the bones were infiltrated and embedded in methyl methacrylate. Nondecalcified samples were further embedded in SCEM media (Section-Lab, C-EM001), from which longitudinal sections of 5-μm thickness were cut using a cryostat (Leica Biosystems, CM3052S) equipped with Clyofilm Type 2C(9) 2.0 cm (Section-Lab). The MAR was determined by dividing the average double-label widths by the designated interlabel duration (4 days for alizarin and 2 days for tetracycline). The BFR was calculated as the product of MAR and the combined lengths of double-labeled and one-half of the single-labeled surfaces, as previously reported (57). For cell identification, Villanueva bone staining was performed, and images were captured under natural light. Osteoclasts were identified using fluorescence microscopy by their adhesion to bone resorptive surfaces and a distinct milky white cytoplasm, with prominent nuclei and peripheral and central chromatin. In contrast, osteoblasts presented with blue cytoplasm and intensely stained nuclei aligned along the osteoid surfaces.

### WB analysis.

Protein samples were prepared by mixing them with Bolt LDS Sample Buffer (Thermo Fisher Scientific, B0007) and denaturing them at 95°C for 5 minutes. Electrophoresis was performed by loading 30 μg of protein per lane on a Bolt 4%–12% Bis-Tris Plus gel (Thermo Fisher Scientific, NW04122BOX) alongside Precision Plus Protein Standards (Bio-Rad, 161-0374) for molecular weight comparison. The proteins were then transferred onto membranes using a semidry method at 20 V for 1 hour. Subsequently, the membranes were blocked in a 5% skim milk solution in PBS for 10 minutes at room temperature. For Piezo1 detection, the membranes were blocked overnight at 4°C. After blocking, the membranes were incubated with primary antibodies in Can Get Signal solution 1 (TOYOBO, NKB-101) at 4°C overnight with the following dilutions: Piezo1 (Proteintech, 15939-I-AP) at 1:300 and Akt (Cell Signaling Technology, 4691), p-Akt (Ser 473; Cell Signaling Technology, 4060), p44/42 MAPK (Erk 1/2; Cell Signaling Technology, 4695), p-p44/42 MAPK (Erk 1/2, Thr202/Tyr204; Cell Signaling Technology, 4370S), CaMKII (Abcam, ab52476), p-CaMKII (Thr286; R&D Systems, PPS002), Hes1 (Adipogen, AG-20T-0400), p-Hes1 (Ser37; Invitrogen, PA 5-105350), cleaved Notch1 (Val1744) (D3B8) (Cell Signaling Technology, 4147S), and β-actin (Cell Signaling Technology, 4970S) at 1:1000. For secondary antibodies, dilutions were made at 1:2000 (1:10,000 for Piezo1) in Can Get Signal Solution 2 (TOYOBO, NKB-101) and incubated for 1 hour at room temperature. Detection was performed using ECL Prime Western Blotting Detection Reagents (Cytiva, RPN2232), and the bands were visualized using a ChemiDoc Imaging System (Bio-Rad). WB analysis was quantified using ImageJ software, and the results were normalized to appropriate standards (Supplemental Figure 18).

### Statistics.

Unless otherwise specified, data were compared between 2 groups using an unpaired, 2-tailed Student’s *t* test (EXSUS ver. 10.0, Eps). For cases involving more than 2 groups, an ANOVA was conducted, followed by a post hoc Tukey-Kramer test (EXSUS ver. 10.0, Eps). *P* values of less than 0.05 were considered statistically significant. Box-and-whisker plots show the median (line within box), IQR (bounds of the boxes), minimum and maximum (whiskers), and outlying value were over third quartile plus (1.5 × IQR), and lower first quartile minus (1.5 × IQR)

### Animals.

The animals were maintained in a controlled environment with a 12-hour light/dark cycle (lights on from 8:00 am to 8:00 pm) at a temperature of 23°C ± 1.5°C and provided with food and water ad libitum. All animal experimental protocols were approved by The Osaka University Animal Care and Use Committee (approval no. 220228).

### Patients.

Between October 2022 and February 2023, a total of 9 patients were enrolled in this study, as shown in Supplemental Table 1. Among these patients, 5 were diagnosed with GIOP, while the remaining 4 had non-GIOP conditions. The patients with GIOP met the criteria outlined in the American College of Rheumatology 2022 Guideline (1), which include having rheumatic or nonrheumatic conditions and being treated with glucocorticoid (prednisolone equivalent ≥ 2.5 mg daily) for more than 3 months. Surgical indications for patients with GIOP included osteonecrosis of the femoral head, with some patients presenting comorbidities such as aortitis syndrome, interstitial pneumonia, dermatomyositis, or systemic lupus erythematosus. On the other hand, patients without GIOP were not administered chronic steroid therapy and did not meet the diagnostic criteria for osteoporosis. Their surgical indications primarily involved osteoarthritis. The study protocol adhered to the ethical principles outlined in the Declaration of Helsinki. Written informed consent was received prior to participation. All clinical procedures were approved by The Clinical Ethics Committee of Osaka University (approval no. 15409).

### Data availability.

The RNA-seq dataset generated from this study has been submitted to the NCBI’s GEO and can be accessed through accession numbers GSE276391 and GSE276529. Additional data supporting the findings of this research are provided within the main text of the article and its Supporting Data Values file. Any further data required can be obtained from the corresponding author upon reasonable request. The source data relevant to this study’s findings are included in the paper.

## Author contributions

NO and KE take responsibility for the integrity of the work as a whole, from inception to the completed manuscript. YE, TN, TM, TK, YF, KU, KT, HH, MT, TI, SI, SY, TK, SO, KN, and KE conceived the study design and performed experiments. YE, TN, TM, TK, YF, SY, and SI analyzed and interpreted data and contributed statistical expertise. TK, SO, KN, and KE provided administrative, technical, or logistic support. KT, HH, MT, and TI contributed key samples. NO and KE wrote the manuscript. All authors edited and approved the manuscript. KN, SO, and KE supervised the study.

## Supplementary Material

Supplemental data

Unedited blot and gel images

Supporting data values

## Figures and Tables

**Table 2:**
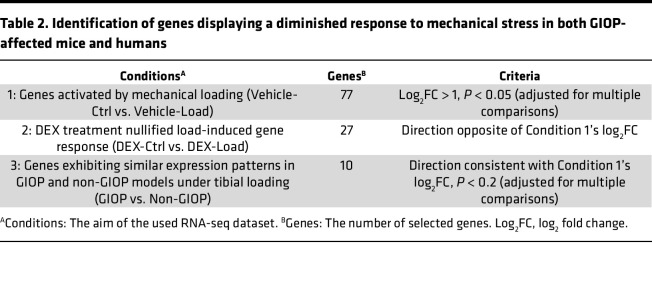
Identification of genes displaying a diminished response to mechanical stress in both GIOP-affected mice and humans

**Table 1:**
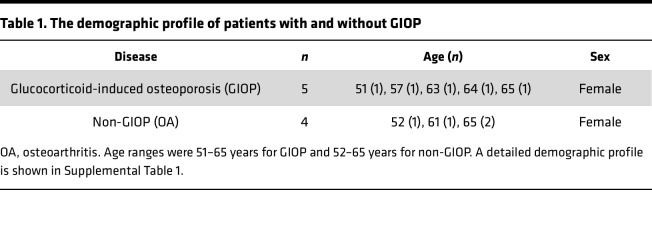
The demographic profile of patients with and without GIOP

**Table 3:**
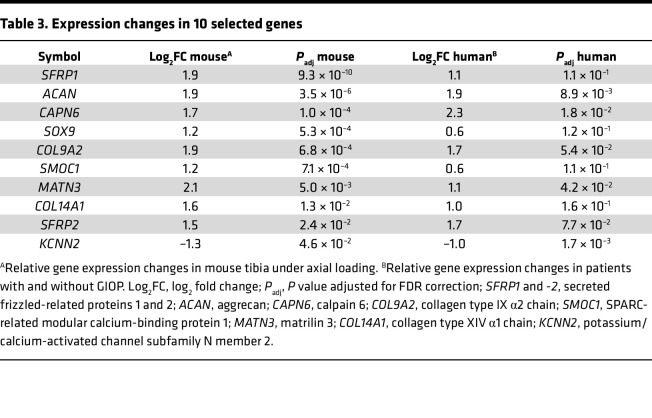
Expression changes in 10 selected genes

## Version 1 12/06/2024

Electronic publication
