# Supplementary material for: The pivotal role of the Hes1/Piezo1 pathway in the pathophysiology of glucocorticoid-induced osteoporosis
Source: JCI Insight. 2024 Dec 6;9(23):e179963. doi: 10.1172/jci.insight.179963 (PMC11623955; doi:10.1172/jci.insight.179963)
Supplement: Supplemental data [file jciinsight-9-179963-s039.pdf]

Supplementary materials

The pivotal role of the Hes1-Piezo1 pathway in the pathophysiology of glucocorticoid-induced osteoporosis

Nagahiro Ochiai, Yuki Etani, Takaaki Noguchi, Taihei Miura, Takuya Kurihara, Yuji Fukuda, Hidetoshi Hamada, Keisuke Uemura, Kazuma Takashima, Masashi Tamaki, Teruya Ishibashi, Shohei Ito, Satoshi Yamakawa, Takashi Kanamoto, Seiji Okada, Ken Nakata, Kosuke Ebina*

***Corresponding author:**

Kosuke Ebina

This file includes:

Supplementary Materials and Methods

Supplementary Figures 1 to 18

Supplementary Tables 1 to 4

Material and Methods

Analysis of Cell Morphology.

MLO-Y4 cells were seeded at 2×10^3 cells/well in collagen-coated 96-well plates (Iwaki, 4860-010) and incubated overnight. The cells were then treated with 1 µM DEX (SIGMA, D1756) and either 1 or 3 µM Yoda1 (Cayman, 21904) for 72 hours. Subsequently, the cells were washed with PBS and fixed using a solution of 4% paraformaldehyde and 0.1% Triton X-100 in PBS for 2 minutes to permeabilize the cell surface. For blocking, a 3% solution of BSA (SIGMA, A2153) in PBS was applied for 30 minutes. F-actin was stained with Rhodamine Phalloidin (Thermo Fisher Scientific, R415), at a final concentration of 66 µM with 0.2% BSA in PBS, and incubated at 4° C overnight. Following PBS washes, the nuclei were stained with 2 µg/mL Hoechst 33258 (Dojindo, H341) in PBS. After a final series of PBS washes, an In Cell Analyzer 6000 (Cytiva) was used to assess cell morphology at 40× magnification.

Electroporation.

MLO-Y4 cells were cultured, harvested using TrypLE Express (Gibco, 12604013), and resuspended in OptiMEM (Gibco, 31985062) to a final concentration of 1×10^6 cells/100 µL. Cells were then combined with either 10 µg of plasmid DNA or 4 pmol of silencer-select siRNA (Thermo Fisher Scientific, Hes1 s67461, Piezo1 s107968, siRNA Negative Control 14390843). This mixture was subjected to electroporation using the NEPA21 system (Nepagene) at 150V with a pulse width of 7.5 msec and a pulse interval of 50 msec.

Calcium Influx Assay.

Piezo1 siRNA or siRNA negative control was electroporated into MLO-Y4 cells, which were then seeded in 96-well plates and incubated overnight. Subsequently, the cells were exposed to 1 µM DEX for 24 hours. After aspiration of the culture medium, 100 µL of αMEM supplemented with 1% FBS was added to each well. According to the Fluo-8 Calcium Flux Assay kit instructions (Abcam, ab112129), 100 µL of Fluo-8 dye loading solution was added to the cells, followed by a 60-minute incubation period. Thereafter, 3 µM Yoda1 was injected using an autosampler, and the fluorescence changes were monitored for 5 minutes using a plate reader (Tristar5, Berthold).

RNA Extraction from Cell Culture.

Cultured cells were rinsed with PBS and lysed using TRIzol reagent (Ambion, 15596018) followed by the addition of chloroform. The lysate was then processed for RNA purification by the Direct-zol MicroPrep kit protocol (Zymo Research, R2052).

Extraction of RNA and Protein from Bone Tissue.

The frozen human or mouse bone tissue was pulverized under liquid nitrogen using a Freezer/Mill 6775 (SPEX). For the extraction of RNA, TRIzol reagent was applied to the crushed tissue, followed by chloroform addition for phase separation. The RNA-containing aqueous phase was isolated and subsequently purified using the Direct-zol MicroPrep kit, as per the manufacturer's instructions. In the case of protein extraction, the crushed tissue was treated with a protein elution buffer (50 mM Tris-HCl, pH 7.5, 0.1% SDS, 250 mM DTT (dithiothreitol) 0.5% NP-40), and the mixture was incubated for 30 minutes at 4° C. After centrifugation at 12,000 g for 10 minutes, the supernatant was collected and served as the protein extract.

Reverse Transcription (RT) Reaction and Quantitative PCR (qPCR).

Reverse transcription was performed using the ReverTra Ace qPCR RT Kit (TOYOBO, FSQ-101) according to the manufacturer's instructions. For qPCR, 8 ng of the RT reaction product was used with the Fast SYBR Green Master Mix (Thermo Fisher Scientific, 4385612) on the StepOne Real-Time PCR System (Thermo Fisher Scientific). The HPRT gene served as the internal control, and relative quantification was carried out using the ΔΔCt method (Supplementary Tables 2 and 3).

TUNEL staining for apoptosis detection.

Following procedures previously documented, both mouse femurs and human femoral neck samples, embedded in paraffin, were sectioned to a thickness of 3 μm and deparaffinized. TUNEL staining was performed using Proteinase K (DAKO, #S3020) treatment for 30 minutes at 37° C. Apoptosis detection was conducted utilizing the MEB STAIN Apoptosis Kit Direct (MBL, #8445) according to the manufacturer's instructions. The percentage of TUNEL-positive cells was quantified through Image J by analyzing merged fluorescent and bright field images.

Histology and immunohistochemistry procedures.

Formalin-fixed, paraffin-embedded, and decalcified sections of the femur and tibia from 3-month-old C57BL/6J male mice, as well as femoral necks from human female patients (Supplementary Table 1), were prepared. Bone specimens were fixed in 4% paraformaldehyde in PBS and decalcified in 20% EDTA. Sequential dehydration in escalating concentrations of ethanol was followed by embedding in paraffin wax. Sections were cut to a thickness of 3 μm for subsequent analyses. For osteocalcin IHC staining, antigen retrieval was executed using proteinase K (DAKO, S3020) for 15 minutes at 37° C. Antigen retrieval for Piezo1 IHC staining involved the use of Tris-EDTA Buffer pH 9.0 (Abcam, ab93684) for 30 minutes at a temperature of 80° C. Endogenous peroxidase activity was suppressed by a 3% sodium hydrogen carbonate solution. Slides were then blocked with Blocking One Hist (Nacalai Tesque, 06349-64), followed by overnight incubation at 4° C with primary antibodies against Osteocalcin (Takara Bio, M188) and Piezo1 (Proteintech, 15939-1), both diluted 1:200 in PBS containing 5% Blocking One Hist. After antibody incubation, sections were washed and incubated with horseradish peroxidase-conjugated secondary antibodies. Signal amplification was achieved using tyramide signal amplification, and visualization employed 3,3′-diaminobenzidine (DAB, Nichirei Bioscience). For Sclerostin (Sost) IHC staining, the ABC system VECTASTAIN ABC kit (Vector Laboratories, PK-4005) protocol was followed, which included a 3% sodium hydrogen carbonate solution to quench endogenous peroxidase activity and blocking with the kit-provided goat serum. Sost antibody (R&D Systems, AF1589) was applied at a dilution of 1:20 in PBS with 1% Triton X-100 for one hour at room temperature. The sections were counterstained with hematoxylin and mounted. Routine hematoxylin and eosin staining and TRAP staining was also carried out. TRAP substrate (Cosmo Bio, AK04F) was used, and according to standard protocols. The number of TRAP-positive cells per trabecular surface in the distal part of the right femurs (1,000 μm width above the growth plate) was determined. Quantitative analyses were conducted using the ImageJ software for counting osteocalcin, Piezo1, Sost-positive cells, and empty lacunae. The ratio of osteocalcin-positive periosteal cells was semiautomatically quantified in Image Scope software (Leica).

Osteoblast differentiation, alkaline phosphatase (ALP) staining, and activity assay, alizarin red S staining.

Human PDCs were plated at a density of 3×10^4 cells per well in collagen-coated 24 well plates with DMEM/F-12 supplemented with 10% FBS and GlutaMAX and incubated overnight. Subsequently, the media was replaced with osteogenic differentiation media from the STEM PRO Osteogenesis Differentiation Kit (Thermo Fisher Scientific, A1007201), renewed every three days. For ALP staining at day 12, cells were washed with PBS and fixed with 4% paraformaldehyde. ALP staining was performed using a solution containing 6.4% nitro blue tetrazolium (NBT), 3.2% 5-bromo-4-chloro-3-indolyl-phosphate (BCIP), 20 mM Tris-HCl at pH 9.0, 40 mM NaCl, and 1 mM $MgCl_2$ (NBT and BCIP sourced from Promega, S3771), with a 10-minute incubation. For the ALP activity assay conducted on day 14, cells were washed with PBS and lysed using M-PER Mammalian Protein Extraction Reagent (Thermo Fisher Scientific, 78501). The ALP activity was measured following the instructions of the LabAssay ALP Kit (Fujifilm, 633-51021). The reaction proceeded for 30 minutes, and the protein concentration was quantified using the Pierce Rapid Gold BCA Protein Assay kit (Thermo Fisher Scientific, A53226) with the results expressed as ALP activity units. For alizarin red S staining at day 21, cells were washed with PBS, fixed with 4% paraformaldehyde, and stained with a 1% alizarin red S solution at pH 6.3-6.4 (Muto Pure Chemicals, 17922) for 10 minutes. Post-staining, mineralization was quantified by eluting the stain in 5% formic acid and measuring the absorbance at 415 nm.

CUT & RUN Assay.

MLO-Y4 cells were electroporated with either Hes1 or a negative control siRNA, followed by overnight incubation. Cells were then exposed to 1 µM DEX and 10 µM Yoda1 for 24 hours. For the CUT & RUN assay, 4×10^5 cells per condition were collected. The assay was performed using the CUT & RUN Assay Kit (Cell Signaling Technology, #86652S), following the manufacturer's instructions. Chromatin immunoprecipitation utilized an anti-Hes1 antibody (Adipogen, AG-20B-0068-c100) and the control sample used the Rabbit (DA1E) mAb IgG XP Isotype Control from the kit, both at a 1:50 dilution for 2 hours at 4° C. For sample normalization, 60 ng of Spike-In DNA (from the kit materials) was added to the concentrated chromatin samples. Samples were then washed, and nucleic acids were purified via phenol/chloroform extraction and ethanol precipitation. The purified DNA was dissolved in TE8.0 (10 mM Tris-HCl, pH 8.0, 1 mM EDTA-2Na, pH 8.0) for qPCR analysis. Primer sequences used for qPCR were included in the supplementary table 4. The results of qPCR were expressed as percent input, calculated using the formula: Percent Input = 100% × 2 ^[C(T) of 100% Input Sample – C(T) of IP Sample].

Luciferase Assay Protocol for MLO-Y4 Cells.

MLO-Y4 cells underwent electroporation with 10 μg of either the pNL3.1[Nluc/minP] Vector (Promega, N1031) or the pNL3.1[Nluc/minP]/Hes1 vector containing a 657 bp Hes1 binding region. For normalization purposes, 1 μg of pGL4.53[Luc2/PGK] Vector (Promega, E5011) was also transfected to the cells, which were then incubated overnight. Post incubation, cells were treated with 1 μM DEX for 24 hours, followed by exposure to 10 μM Yoda1 for an additional 4 hours. The luciferase assay was conducted using the Nano-Glo Dual-Luciferase Reporter Assay System (Promega, N1610) as followed by the manufacturer's protocol.

Protein Extraction from Cultured MLO-Y4 Cells.

MLO-Y4 cells were plated at a density of 3×10^5 cells/well on type-I collagen-coated 6-well plates (Iwaki, #4810-010) and incubated overnight. The cells were then treated with 1 μM DEX for 24 hours, followed by variable exposures to 10 μM Yoda1 ranging from 1 to 24 hours depending on the protein targets of interest. For CaM kinase II inhibition, cells were pre-incubated with either 1 μM or 3 μM KN93 (Cayman Chemical, #21472) for 2 hours. After washing with PBS, the cells were lysed using RIPA buffer (Thermo Fisher Scientific) supplemented with a 1% Protease/Phosphatase Inhibitor Cocktail (100×) (Cell Signaling Technology, #5872). Lysates were then sonicated using a Bioruptor II (Cosmo Bio), and centrifuged at 12,000 g for 5 minutes. The resulting supernatants had their protein concentrations determined using the Pierce Rapid Gold BCA Protein Assay Kit (Thermo Fisher Scientific, A53225).

References

1. Hilton MJ, ed. *Skeletal Development and Repair: Methods and Protocols*. New York, NY: Springer US; 2021.

2. Zou Z, et al. ChIP-Atlas 2021 update: a data-mining suite for exploring epigenomic landscapes by fully integrating ChIP-seq, ATAC-seq and Bisulfite-seq data. *Nucleic Acids Research*. 2022;50(W1):W175–W182.

3. Oki S, et al. ChIP - Atlas: a data - mining suite powered by full integration of public ChIP - seq data. *EMBO reports*. 2018;19(12):e46255.

4. Robinson JT, et al. Integrative genomics viewer. *Nat Biotechnol*. 2011;29(1):24–26.

5. Thorvaldsdóttir H, Robinson JT, Mesirov JP. Integrative Genomics Viewer (IGV): high-performance genomics data visualization and exploration. *Briefings in Bioinformatics*. 2013;14(2):178–192.

6. Robinson JT, et al. Variant Review with the Integrative Genomics Viewer. *Cancer Research*. 2017;77(21):e31–e34.

7. Robinson JT, et al. igv.js: an embeddable JavaScript implementation of the Integrative Genomics Viewer (IGV). *Bioinformatics*. 2023;39(1):btac830.

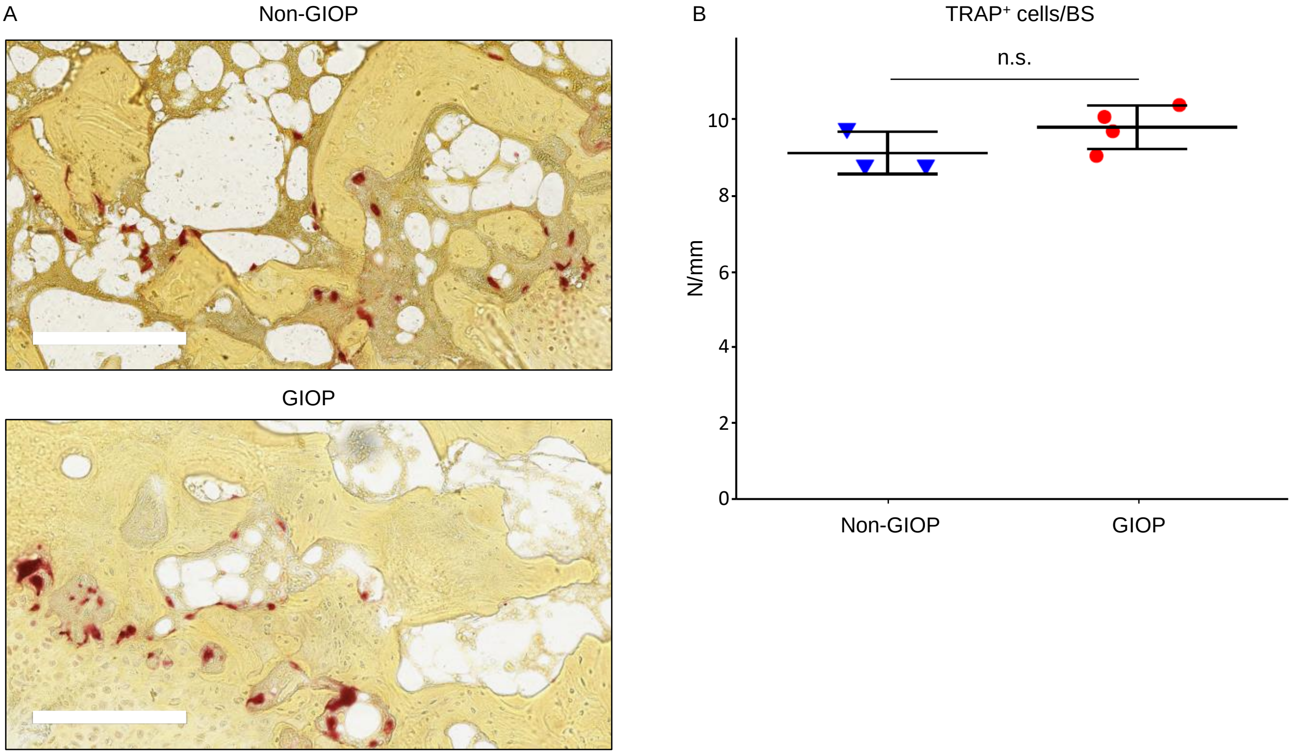

Supplementary Figure 1: Comparative analysis of TRAP-positive cells between non-GIOP and GIOP subjects.
A: TRAP staining was performed on femur samples obtained from patients with non-glucocorticoid-induced osteoporosis (Non-GIOP) and glucocorticoid-induced osteoporosis (GIOP). B: The quantification of TRAP-positive cells at the bone surface is presented. Data are expressed as the mean ± SD for each group (Non-GIOP, n=3, GIOP, *n* = 4). Statistical significance was assessed using the Student's *t*-test not significant (n.s.) difference was observed between the two groups.

.

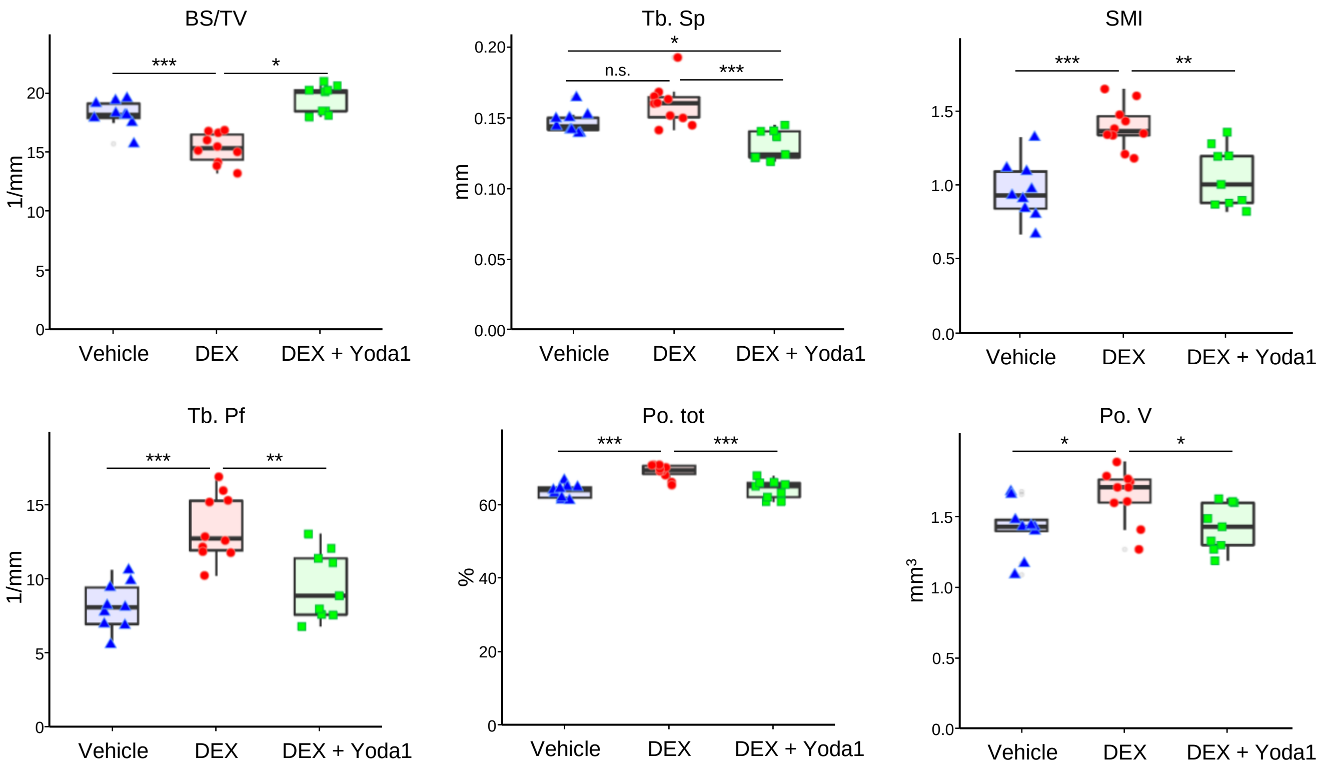

Supplementary Figure 2: Micro CT analysis of DEX and Yoda1 treated mice.
DEX: DEX 1mg/kg s.c. , Yoda1: 5 µmol/kg i.p., Vehicle: water for injection s.c. as for DEX and 5% ethanol i.p. as for Yoda1. Each condition was 5 times injections within one week for 4 weeks. The groups in the study were: Vehicle (vehicle-treated), DEX (DEX-treated), and DEX + Yoda1 (concomitant administration of DEX and Yoda1). BS/TV (bone surface to tissue volume ratio), Tb. Sp (trabecular separation), SMI: structure model index, Tb. Pf: Trabecular bone pattern factor, Po. tot.: Total porosity, Po. V: Total pore volume. Data are expressed as box-and-whisker plots for each group (n = 9). Statistical significance was assessed using one-way ANOVA, the Tukey-Kramer test, *$p < 0.05$, **$p < 0.01$, ***$p < 0.001$. n.s.: not significant.

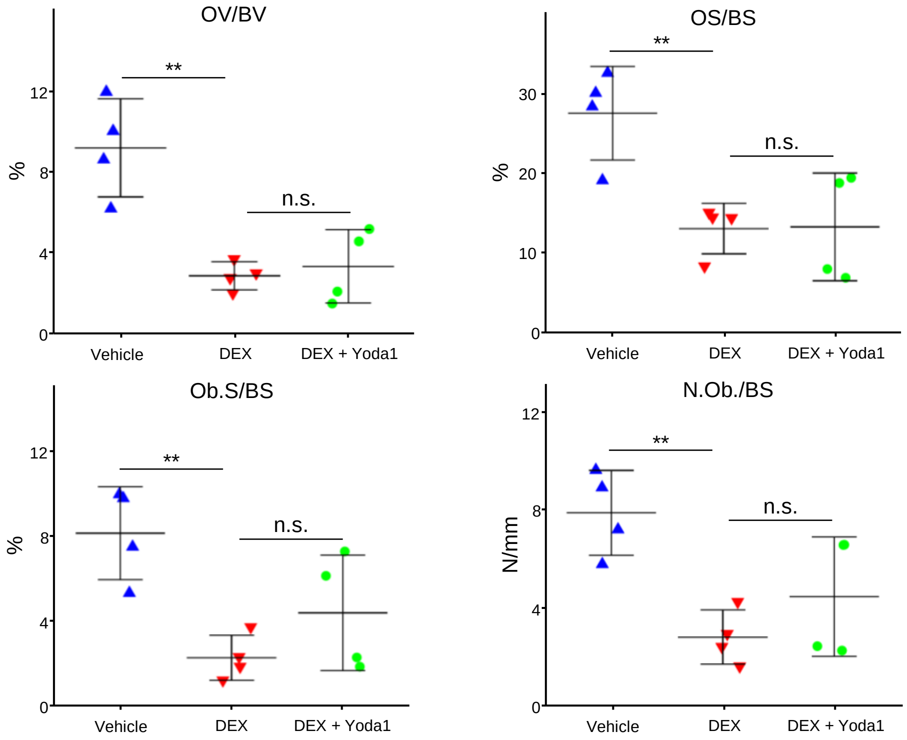

Supplementary Figure 3: Bone histomorphometry of DEX and Yoda1 Treated Mice.

DEX: 1 mg/kg s.c., Yoda1: 5 µmol/kg i.p., Vehicle: Water for injection s.c. for DEX and 5% ethanol i.p. for Yoda1. Each treatment was administered five times per week for four weeks. The study groups included: Vehicle (vehicle-treated), DEX (DEX-treated), and DEX + Yoda1 (concomitant administration of DEX and Yoda1). The measured parameters were OV/BV (osteoid volume/bone volume), OS/BS (osteoid surface/bone surface), Ob.S/BS (osteoblast surface/bone surface), and N.Ob./BS (number of osteoblasts/bone surface). Data are expressed as the mean ± SD for each group ($n = 4$). Statistical significance was assessed using one-way ANOVA followed by the Tukey-Kramer test, **$p < 0.01$, n.s.: not significant.

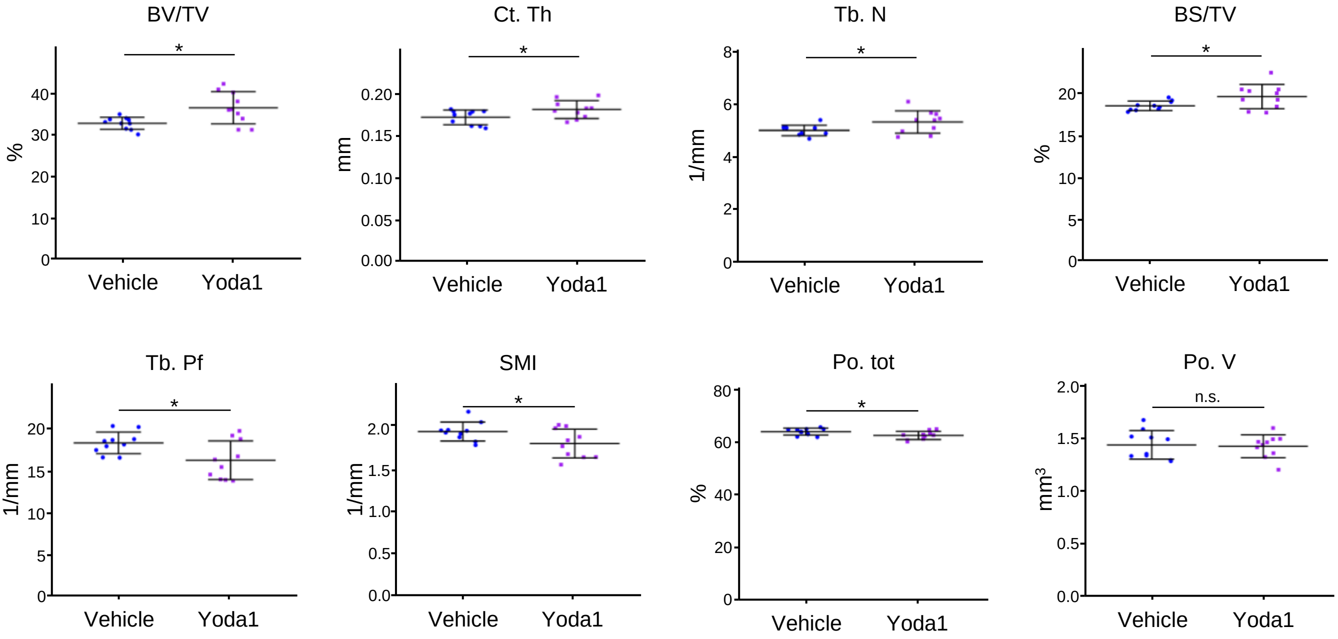

Supplementary Figure 4: Micro-CT Analysis of Yoda1 Treated Mice
Yoda1 (5 µmol/kg i.p.), Vehicle (water with 5% ethanol i.p.). Treatments were administered five times per week for four weeks. BV/TV (bone volume/tissue volume), Ct. Th (cortical thickness), Tb. N (trabecular number), BS/TV (bone surface/tissue volume), Tb. Pf (trabecular bone pattern factor), SMI (structure model index), Po. tot. (total porosity), Po. V (total pore volume). Data are expressed as mean ± SD (n = 10). Statistical analysis was performed using a two-tailed Student's t-test with a 95% confidence interval. *$p < 0.05$, n.s.: not significant.

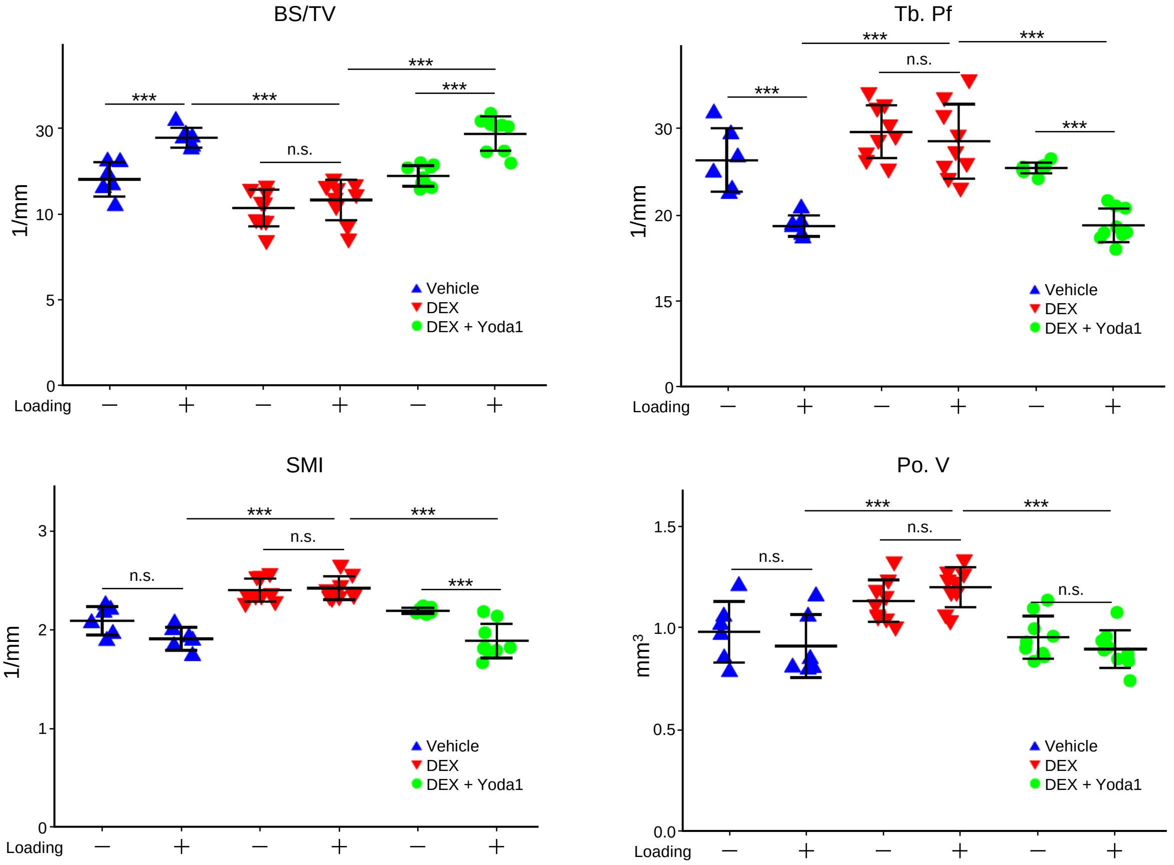

Supplementary Figure 5: Micro CT analysis of tibia axial loading for DEX and Yoda1 treated mice.
DEX: DEX 1mg/kg s.c. , Yoda1: 5 µmol/kg i.p., Vehicle: water for injection s.c. as for DEX and 5% ethanol i.p. as for Yoda1. Each condition was 5 times injections within one week. The conditions for tibial axial loading were set for the left tibia, which was loaded 2-3 times per week, with sessions of 40 cycles per day, using a trapezoidal waveform at a force of -13N for 0.1 seconds with an interval of 10 seconds each cycle. Loading was under the control of Electroforce 5500. The groups in the study were: Vehicle (vehicle-treated), DEX (DEX-treated), and DEX + Yoda1 (concomitant administration of DEX and Yoda1). BS/TV (bone surface to tissue volume ratio), SMI: structure model index, Tb. Pf: Trabecular bone pattern factor, Po. V: Total pore volume. Data are expressed as box-and-whisker plots for each group (n = 6-9). Statistical significance was assessed using one-way ANOVA, the Tukey-Kramer test, ***$p < 0.001$. n.s.: not significant.

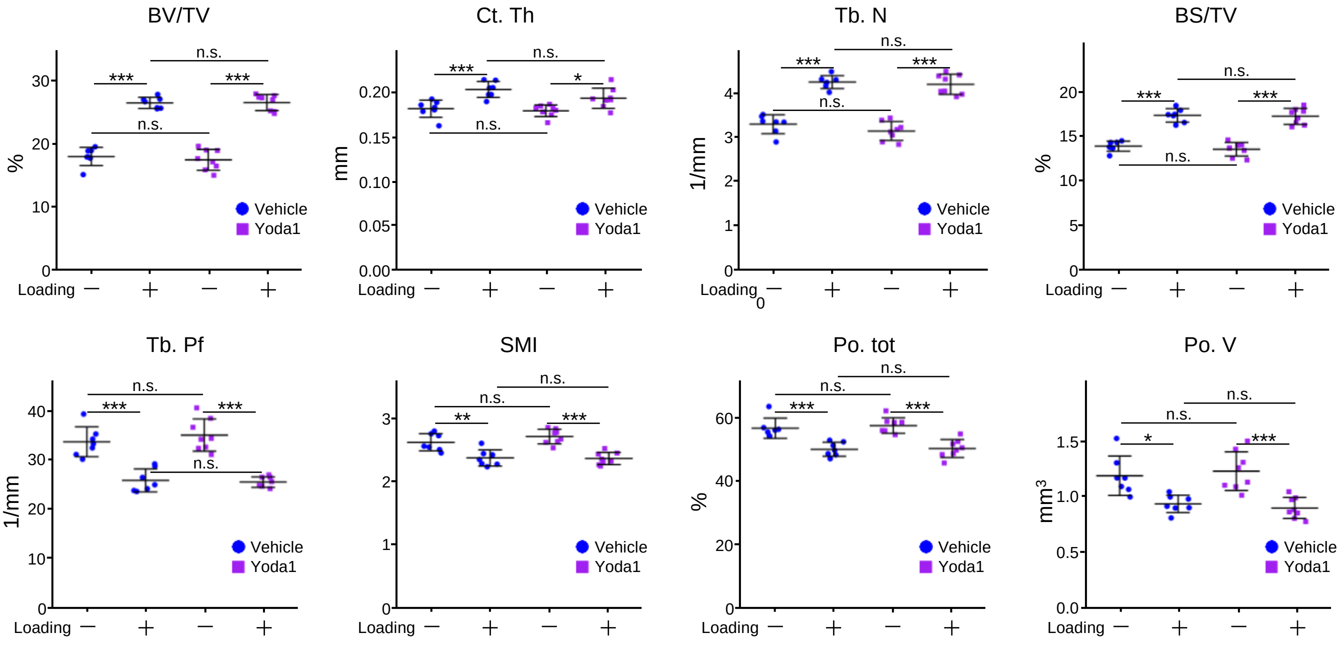

Supplementary Figure 6: Micro-CT Analysis of Tibia Axial Loading in Yoda1 Treated Mice
Yoda1 (5 μmol/kg i.p.), Vehicle (water with 5% ethanol i.p.). Treatments were administered five times per week. Tibial axial loading was applied to the left tibia 2-3 times per week, with sessions of 40 cycles per day. Each cycle used a trapezoidal waveform at a force of -13N for 0.1 seconds, with a 10-second interval between cycles. Loading was controlled using an Electroforce 5500. BV/TV (bone volume/tissue volume), Ct. Th (cortical thickness), Tb. N (trabecular number), BS/TV (bone surface/tissue volume), Tb. Pf (trabecular bone pattern factor), SMI (structure model index), Po. tot. (total porosity), Po. V (total pore volume). Data are expressed as mean ± SD (n = 7-8). Statistical significance was assessed using one-way ANOVA with the Tukey-Kramer test. *p < 0.05, **p < 0.01 , ***p < 0.001, n.s.: not significant.

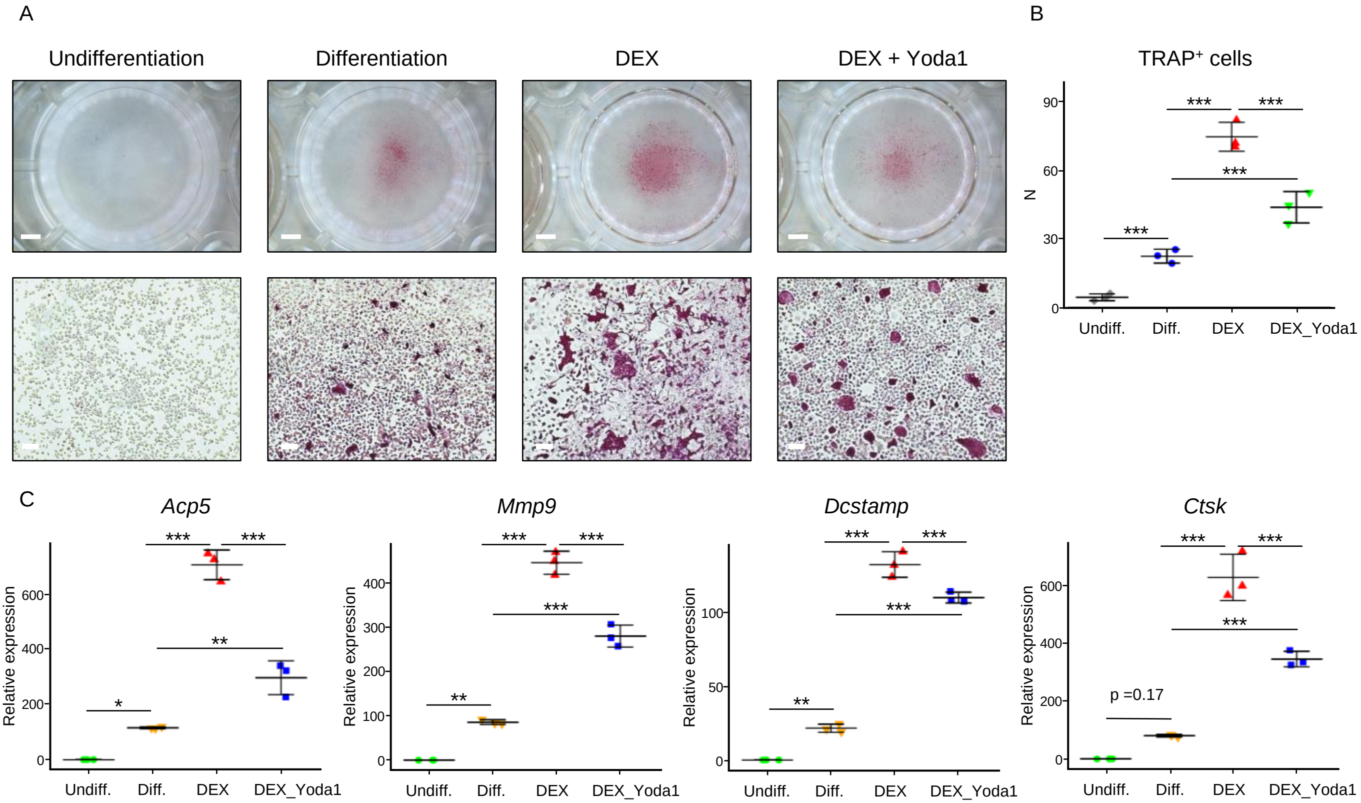

Supplementary Figure 7: Partial suppression of osteoclastogenesis by combined administration of DEX and Yoda1. Bone marrow-derived macrophages (BMDMs), harvested from murine femurs, were cultured in 24-well plates with macrophage colony-stimulating factor (M-CSF) at 30 ng/mL for 3 days to promote survival. To induce osteoclast differentiation, RANKL at 50 ng/mL was added, along with simultaneous treatment of DEX (1 µM) and Yoda1 (1 µM) in the respective wells. After 7 days, TRAP staining was executed, followed by RNA extraction for qPCR The grouping for the experiment was as follows: Undiff. (undifferentiated BMMs treated with M-CSF), Diff. (osteoclast differentiation induced with M-CSF and RANKL), DEX (BMDMs treated with M-CSF, RANKL, and DEX), and DEX-Yoda1 (BMDMs treated with M-CSF, RANKL, DEX, and Yoda1). (**A**) TRAP-stained images were captured at a low-power field with objective magnification at ×1.25 (scale bar: 2 mm), and at a high-power field with objective magnification at × 10 (scale bar: 100 µm). (**B**) Quantification of TRAP-positive multinucleated cells. (**C**) Changes in the expression of osteoclast-specific markers were analyzed by qPCR. Relative expression levels were determined using the ΔΔCt method, with Hprt serving as an internal control. Gene markers analyzed were Acp5 (acid phosphatase 5, tartrate-resistant), Mmp9 (matrix metalloproteinase-9), Dcstamp (dendritic cell-specific transmembrane protein), and Ctsk (cathepsin K). Data are presented as means ± SD, with a sample size of n = 3 for each group. Statistical significance was evaluated using one-way ANOVA with the Tukey-Kramer post hoc test, with the significance levels indicated by *p < 0.05, **p < 0.01, ***p < 0.001.

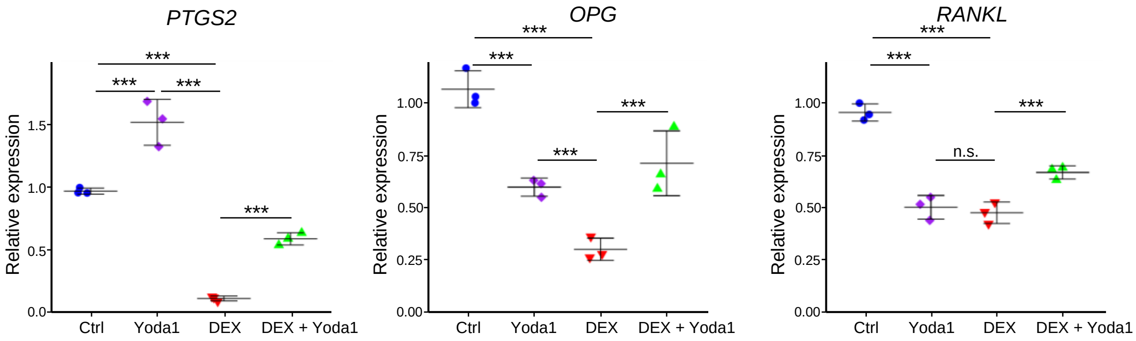

Supplementary Figure 8: The effects of DEX and Yoda1 on human cortical bone.
Cleared of soft tissue and periosteum, human femoral neck cortical bone samples obtained from hip arthroplasty procedures were subjected to an overnight incubation with DEX, followed by a 6-hour treatment with Yoda1 (n = 3). The experimental groups were as follows: Control (no treatment), Yoda1 alone (10 μM), DEX alone (1 μM), and DEX + Yoda1 (concomitant administration of 1 μM DEX and 10 μM Yoda1). Data are expressed as mean ± SD. Statistical analyses were conducted using one-way ANOVA with the Tukey-Kramer post hoc test for multiple comparisons. Significance levels are denoted by ***p < 0.001 , n.s.: not significant..

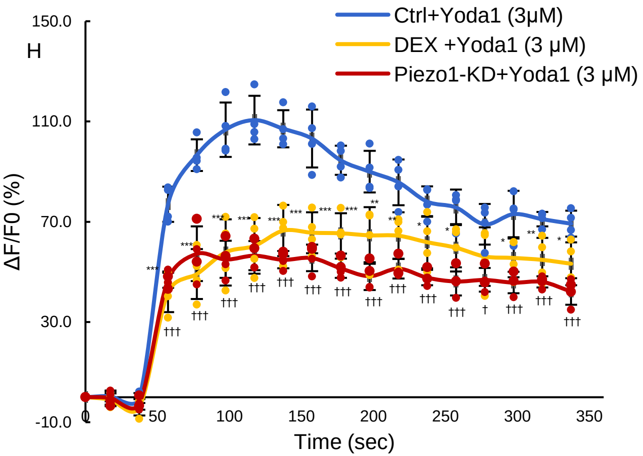

Supplementary Figure 9: Yoda1-stimulated Ca^{2+} influx and its suppression by DEX.
MLO-Y4 cells were transfected with either Piezo1 siRNA or control RNA, followed by treatment with DEX (1 µM) for 24 hours. Subsequently, all groups were treated with Yoda1 (3 µM) to monitor changes in Ca^{2+} influx ($n = 4$). Results are presented as mean ± SD. Statistical significance was assessed using one-way ANOVA followed by Tukey-Kramer post-hoc test. Statistical significance is indicated as *$p < 0.05$, **$p < 0.01$, and ***$p < 0.001$, † < 0.05, ††< 0.01, and ††† < 0.001. *, Control vs. DEX; †, Control vs. Piezo1-knockdown.

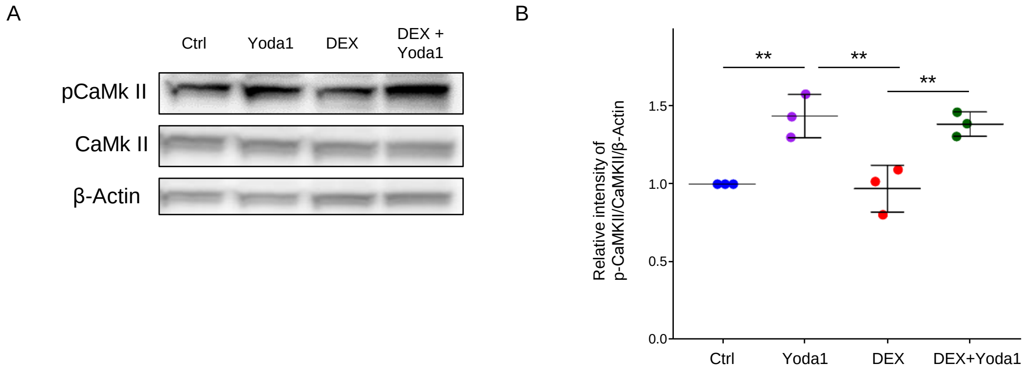

Supplementary Figure 10: Yoda1 activated CaM kinase II phosphorylation
(**A**) Western blot analysis of CaMKII phosphorylation in MLO-Y4 cells treated with DEX, followed by 1-hour exposure to Yoda1 (10 μM). Three experimental groups were established: Control (untreated), DEX alone (1 μM), and DEX+Yoda1 (combination of 1 μM DEX and 10 μM Yoda1). (**B**) Quantification of the Western blot results using ImageJ (*n* = 3). The intensity of the bands was normalized to CaM kinase II and β-Actin. Statistical significance was assessed using one-way ANOVA with the Tukey-Kramer test. $^{**}p < 0.01$.

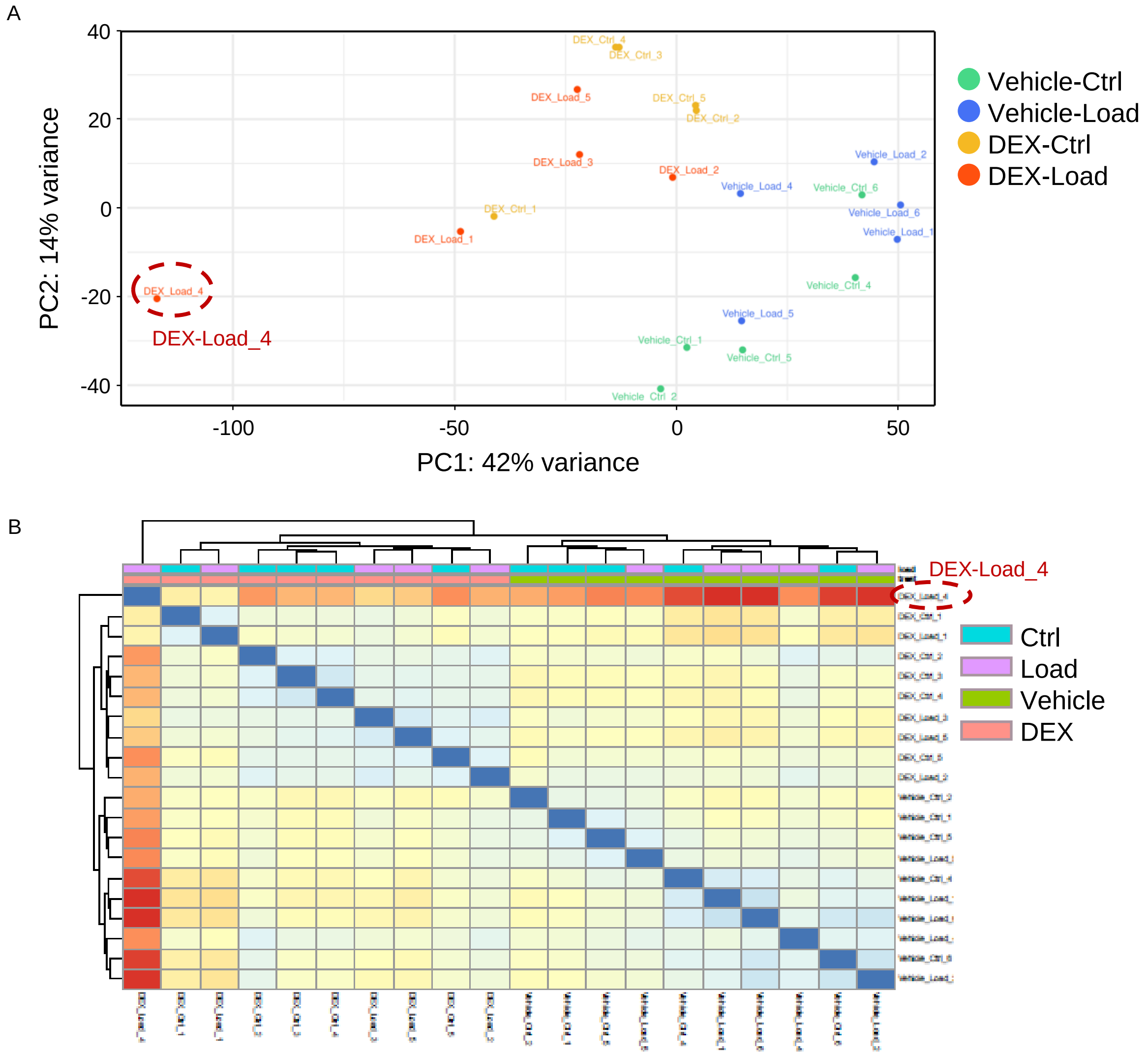

Supplementary Figure 11: Quality control of RNA sequencing analysis.
Experimental groups are labeled as follows: Vehicle (injection with water), DEX (dexamethasone at 1 mg/kg administered), Ctrl (right tibia not subjected to mechanical load), and Load (left tibia subjected to mechanical load). (**A**) Principal Component Analysis (PCA) revealed that the sample DEX-Load_4 exhibits a distinct clustering pattern separate from the other samples. (**B**) Hierarchical cluster analysis demonstrates the grouping of the samples, with DEX-Load_4 being identified as an outlier.

A

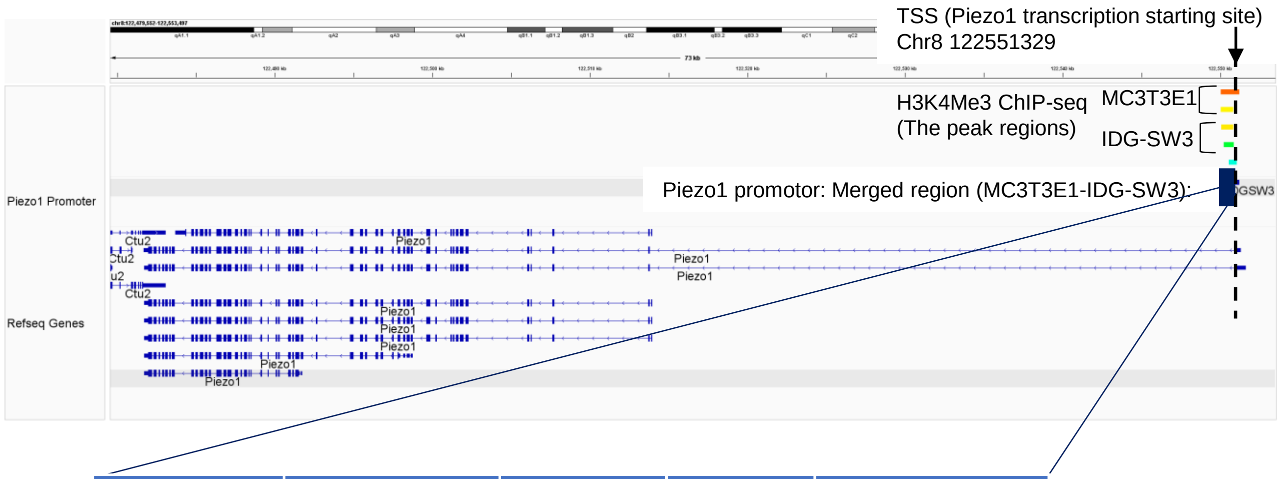

B

Chromosome	Start	End	Width	Name
chr8	122550048<br>(-1281 from TSS)	122551247<br>(-82)	1200	Merged region<br>(Piezo1 promoter)

C

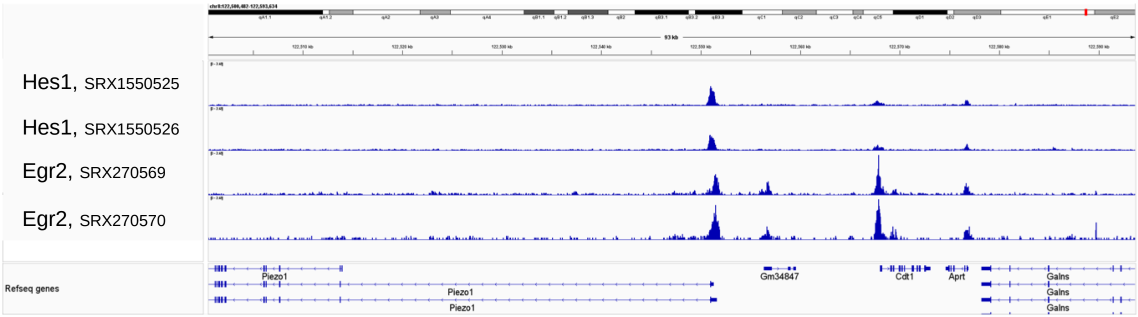

Supplementary Figure 12: Identification of potential transcription factors for Piezo1.
(**A**) For the selection of potential transcription factor candidates for Piezo1, we retrieved trimethylation of lysine 4 on histone H3 (H3K4Me3) chromatin immunoprecipitation sequencing (ChIP-seq) data. This dataset was obtained for the osteoblast/osteocyte-established cell lines MC3T3-E1 and IDG-SW3. We defined the promoter region of Piezo1 by integrating signals from both cell lines. (**B**) We determined the start and end positions of the Piezo1 promoter based on the combined H3K4Me3 ChIP-seq signal enrichment. (**C**) Transcriptomic analysis was conducted by obtaining ChIP-seq datasets for various transcription factors, such as Hes1 and Egr2. We selected those factors exhibiting binding peaks proximal to the Piezo1 promoter region. From this analysis, we identified 145 candidate genes that may regulate Piezo1 expression. All utilized ChIP-seq datasets were retrieved from the ChIP Atlas (https://chip-atlas.org/) (2, 3).

A

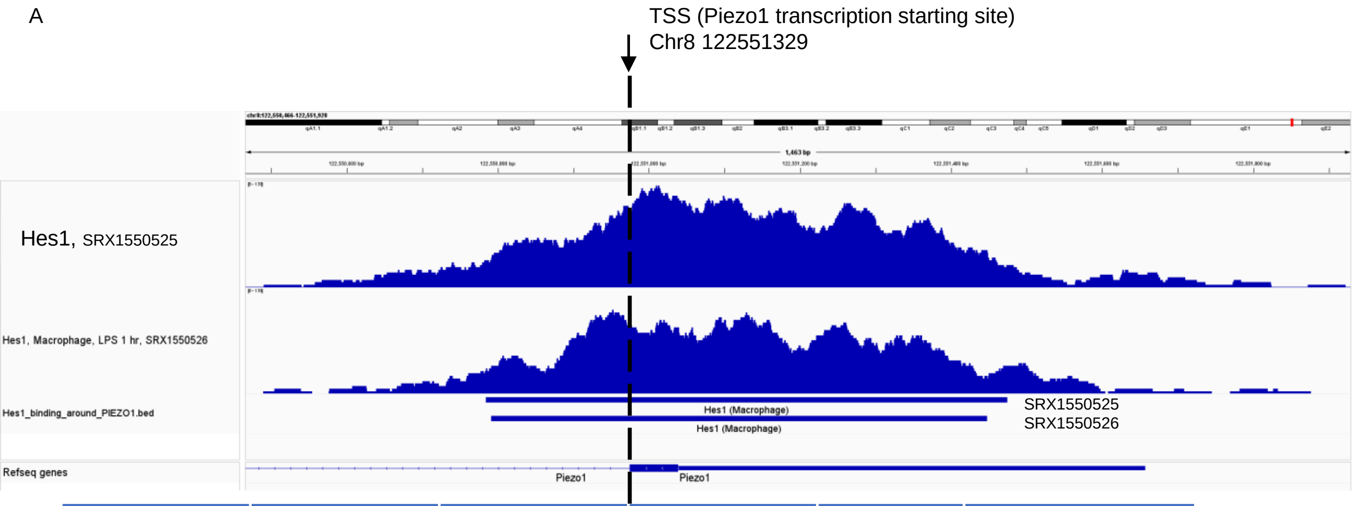

B

SRX	Chromosome	Start	End	Width	Name
SRX1550525	Chr8	122550791 (-538)	122551447 (+118)	657	Hes1 binding region
SRX1550526	Chr8	122550784 (-545)	122551474 (+145)	691	

Supplementary Figure 13: Determination of Hes1 binding region.
To identify the specific Hes1 protein binding sites around the Piezo1 promoter, the following approach was taken: (**A**) We utilized the Integrative Genomics Viewer (IGV, https://igv.org/) to visualize potential Hes1 binding regions upstream of the Piezo1 TSS (4-7). (**B**) Analysis of Hes1 ChIP-seq datasets (study IDs: SRX1550525, SRX1550526) revealed two segments with significant peak enrichment. We confirmed the intersecting sequence between these peaks (-538 bp to +118 bp relative to the Piezo1 TSS) as the likely Hes1 binding domain.

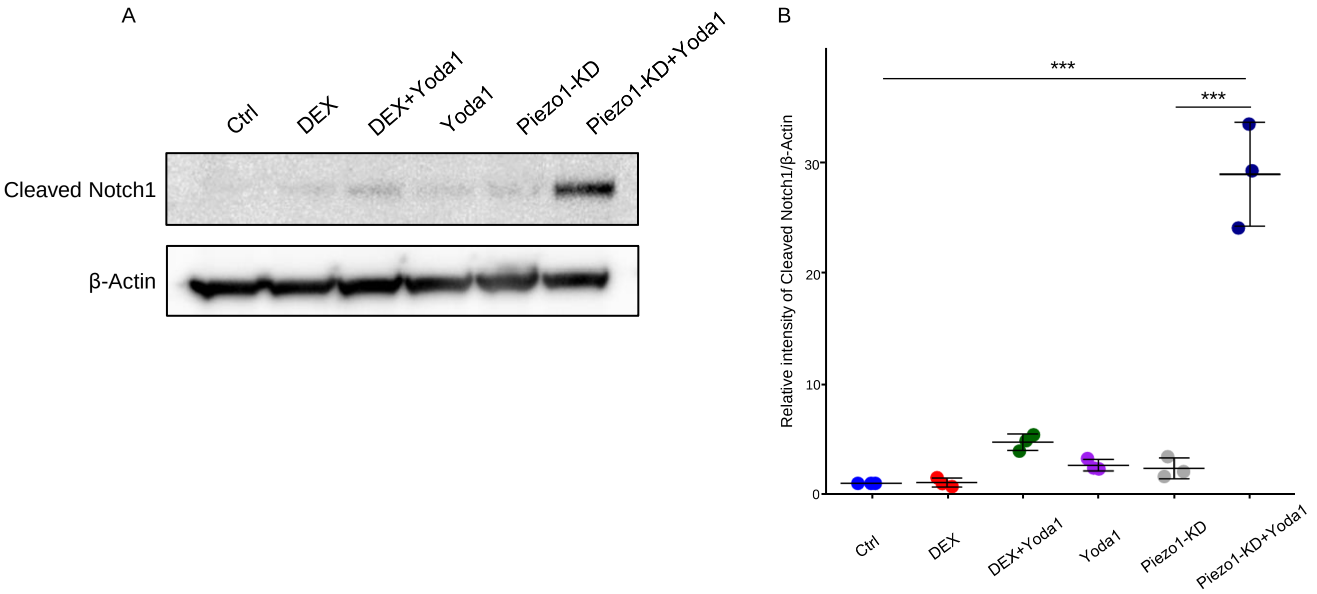

Supplementary Figure 14: Yoda1 activated Notch signaling through cleaved Notch1.
(**A**) Western blot analysis of cleaved Notch1. MLO-Y4 cells were transfected with either Piezo1 siRNA (Piezo1-KD) or control RNA, followed by treatment with DEX (1 µM) for 24 hours. Subsequently, the cells were treated with Yoda1 (10 µM) for 0.5 hours. (**B**) Quantification of the Western blot analysis using ImageJ ($n = 3$). The intensity of the bands was normalized to β-Actin. Statistical significance was assessed using one-way ANOVA with the Tukey-Kramer test. ***$p < 0.001$.

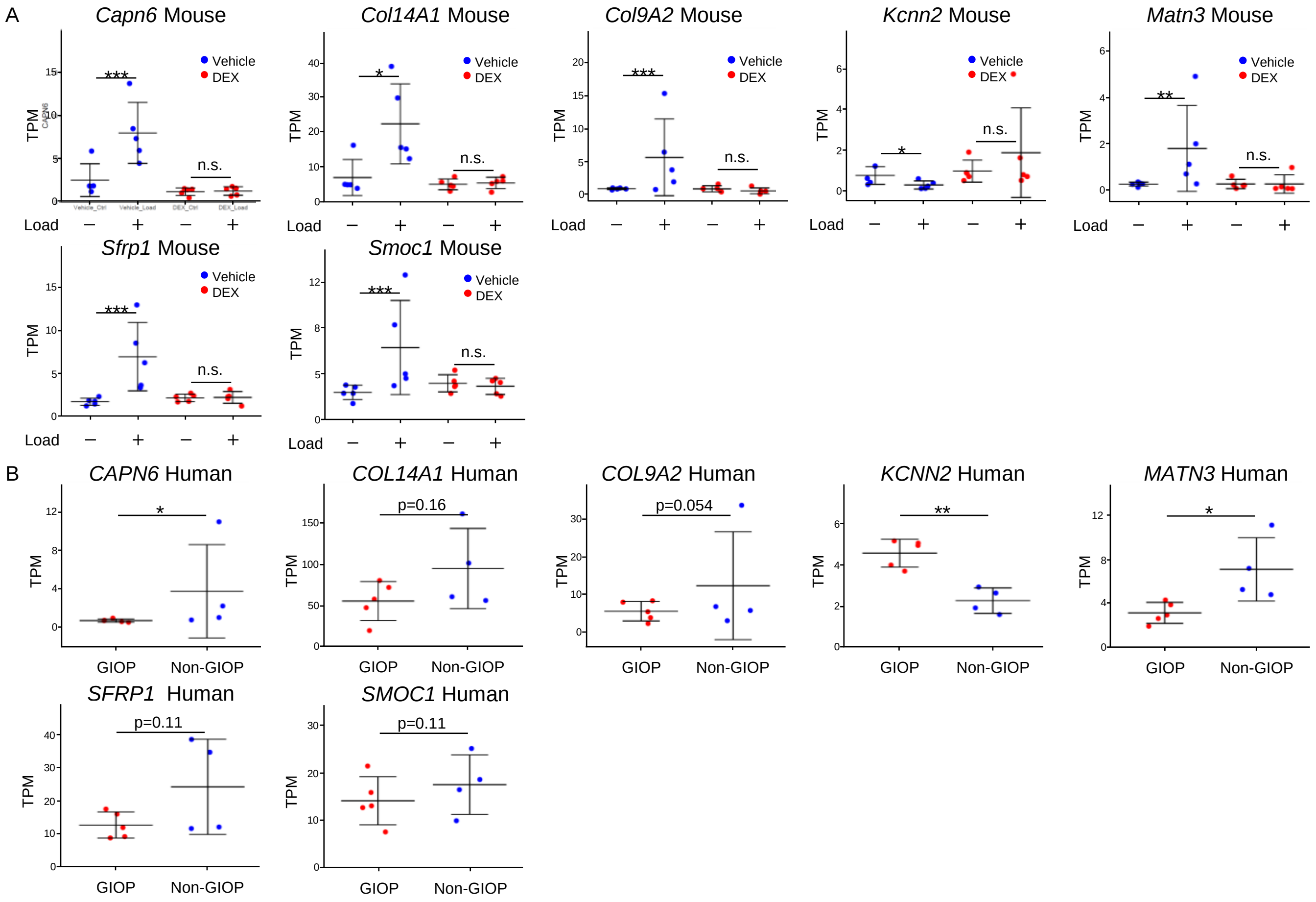

Supplementary Figure 15: Alterations in gene expression in mouse tibial loading and human GIOP. (**A**) Gene expression profiling of mouse tibia following mechanical loading was obtained through RNA sequencing. DEX was administered s.c. at a dose of 1 mg/kg (DEX group), while a vehicle control group received distilled water injections (Vehicle group). Both treatments involved five injections over the course of one week. The left tibia was subjected to axial loading three times per week, with each session consisting of 40 cycles (trapezoidal waveform) at a force of 13N for 0.1 seconds, with a 10-second interval between cycles. This loading regimen was conducted using an ElectroForce 5500 system. Four hours after the last loading session on day five, mice were sacrificed, and their tibiae were promptly flash-frozen in liquid nitrogen for subsequent RNA extraction and sequencing. (**B**) Assessment of the cortical bone RNA profiles in GIOP versus non-GIOP conditions was performed, with reference definitions for these conditions provided in Supplementary Table 1. CAPN6 (Calpain 6), COL14A1 (Collagen Type XIV Alpha 1 Chain), COL9A2 (Collagen Type IX Alpha 2 Chain), KCNN2 (Potassium Calcium-Activated Channel Subfamily N Member 2), MATN3 (Matrilin 3), SFRP1 (Secreted Frizzled Related Protein 1), and SMOC1 (SPARC-Related Modular Calcium Binding 1). Data are expressed as mean ± SD, with n = 4-5 mice per group. Statistical tests were performed using the Wald test according to the DESeq2 workflow and using the proper statistical model. Levels of significance were denoted as *$p < 0.05$, **$p < 0.01$, ***$p < 0.001$, n.s.: not significant, with the false discovery rate (FDR) correction applied to account for multiple error risks.

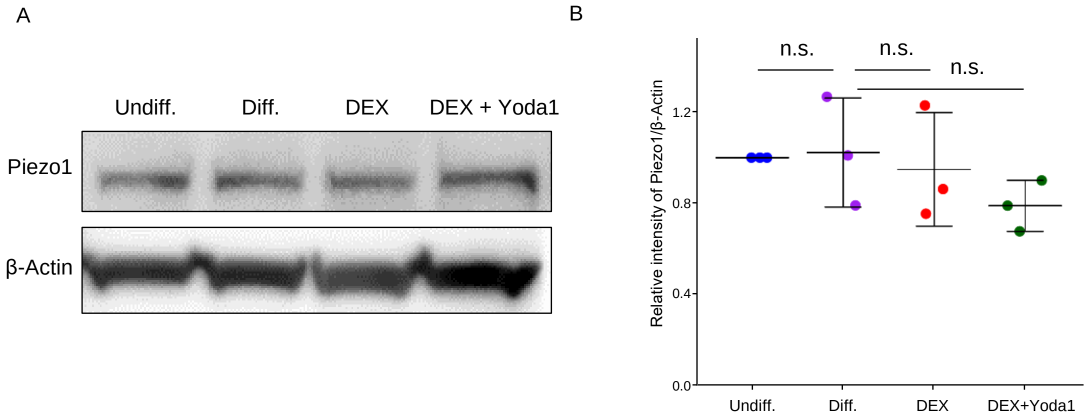

Supplementary Figure 16: Piezo1 expression in 3T3-E1 cells during osteoblast differentiation and the effects of DEX and Yoda1. (**A**) MC3T3-E1 cells underwent osteoblast differentiation with ascorbic acid at 50 μg/mL and β-glycerophosphate at 10 mM for three days. Post-differentiation, the culture medium was replaced, and cells were treated with 1 μM DEX overnight, followed by a 4-hour exposure to 10 μM Yoda1 before protein extraction for Western blotting. The groups included undifferentiated 3T3-E1 cells (Undiff.), osteoblast-differentiated 3T3-E1 cells (Diff.), cells treated with DEX after differentiation (DEX), and cells treated with DEX followed by Yoda1 after differentiation (DEX + Yoda1) (**B**) Quantification of the Western blot analysis using ImageJ (*n* = 3). Band intensities were normalized to β-Actin. Statistical significance was assessed using one-way ANOVA with the Tukey-Kramer test. n.s.: not significant.

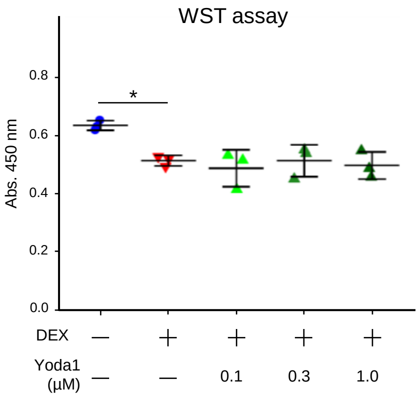

Supplementary Figure 17: WST (Water soluble tetrazolium) assay for PDCs.
Cell viability post-osteoblast induction was assessed by WST assay after 24 hours, following concurrent treatments with DEX (1 µM) and Yoda1 (0.1, 0.3, 1.0 µM) for 24 hours, monitoring with absorbance measure at 450 nm (n = 3). Data are presented as mean ± SD. One-way ANOVA coupled with the Tukey-Kramer post-hoc test was employed for statistical analysis. Significance designated by *$p < 0.05$.

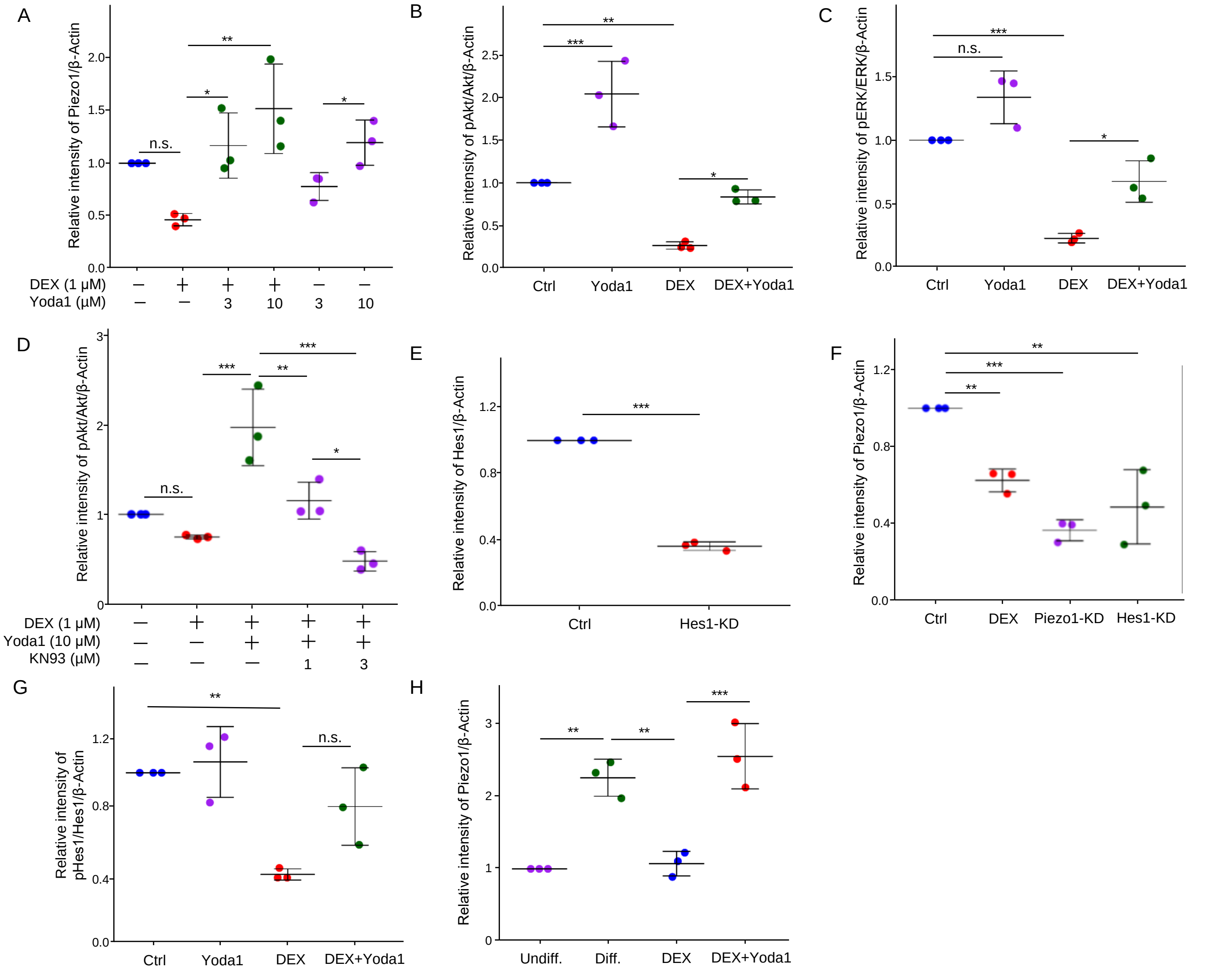

Supplementary Figure 18: Quantification of Western blot analysis

Quantification of the Western blot analysis using ImageJ. (**A**) Corresponding to Figure 5E: Band intensities were normalized to β-Actin. (**B**) Corresponding to Figure 5F: Band intensities were normalized to Akt and β-Actin. (**C**) Corresponding to Figure 5G: Band intensities were normalized to ERK and β-Actin. (**D**) Corresponding to Figure 5J: Band intensities were normalized to Akt and β-Actin. (**E**), (**F**) Corresponding to Figure 7B: Band intensities were normalized to β-Actin. (**G**) Corresponding to Figure 7H: Band intensities were normalized to Hes1 and β-Actin. (**H**) Corresponding to Figure 9A: Band intensities were normalized to β-Actin. Data are expressed as mean ± SD (n = 3). Statistical significance was assessed using one-way ANOVA with the Tukey-Kramer test. (**E**) A two-tailed Student's t-test with a 95% confidence interval. *$p < 0.05$, **$p < 0.01$, ***$p < 0.001$, n.s.: not significant.

Supplementary Table 1: Clinical background of patients with non-glucocorticoid-induced osteoporosis (non-GIOP) and glucocorticoid-induced osteoporosis (GIOP).

Age	Sex	ID	Medication history for osteoporosis	Steroid usage history	Present illness	Lumbar spine (g/cm^2)	Lumbar spine (T-score)	Total Hip (g/cm^2)	Total Hip (T-score)
65	F	Non-GIOP 1	Not applicable	Not applicable	Osteoarthritis (OA)	0.938	-0.6	0.760	-0.3
65	F	Non-GIOP 2	Not applicable	Not applicable	Osteoarthritis (OA)	0.893	1.0	0.629	-1.8
61	F	Non-GIOP 3	Not applicable	Not applicable	Osteoarthritis (OA)	0.908	0.9	0.729	-0.7
52	F	Non- GIOP 4	Not applicable	Not applicable	Osteoarthritis (OA)	1.235	1.9	0.764	-0.3
65	F	GIOP 1	Alendronate	>5 years, prednisolone 4 mg	Aortitis Syndrome	0.780	-1.9	0.694	-1.1
63	F	GIOP 2	Denosumab	>5 years, prednisolone 5 mg	Interstitial pneumonia (IP), Dermatomyositis	1.110	0.8	0.614	-2.0
57	F	GIOP 3	Alendronate	>5 years, prednisolone 4 mg	Systemic lupus erythematosus (SLE)	0.919	-0.8	0.752	-0.4
51	F	GIOP 4	Denosumab	>5 years, methylprednisolone 4mg	Systemic lupus erythematosus (SLE)	1.071	0.5	0.681	-1.2
64	F	GIOP 5	Eldecalcitol Minodronic Acid Hydrate	>5 years, prednisolone 5mg	Interstitial pneumonia (IP)	1.035	0.20	0.758	-0.4

Age range: non-GIOP (52–65 years), GIOP (51–65 years). F denotes female. Bone mineral density of the lumbar spine (L2-4) and total hip were assessed one month before total hip arthroplasty surgery.

Supplementary Table 2: Primer sequences for qPCR of mouse genes.

Gene (Mouse)	Direction	Primer Sequence (5' to 3')
Hprt1	Forward	CTGGTGAAAAGGACCTCTCGAA
Hprt1	Reverse	CTGAAGTACTCATTATAGTCAAGGGCAT
Piezo1	Forward	GTTACCCCCTGGGAACATCT
Piezo1	Reverse	TTCAGGAGAGAGGTGGCTGT
Hes1	Forward	GGAAATGACTGTGAAGCACCTCC
Hes1	Reverse	GAAGCGGGTCACCTCGTTCATG
Wnt16	Forward	GGAGCTGTGCAAGAGGAAAC
Wnt16	Reverse	AGTGGCGACCATACAGTTCC
Tnfrsf11b (Opg)	Forward	CGGAAACAGAGAAGCCACGCAA
Tnfrsf11b (Opg)	Reverse	CTGTCCACCAAAACACTCAGCC
Tnfrsf11 (Rankl)	Forward	GTGAAGACACACTACCTGACTCC
Tnfrsf11 (Rankl)	Reverse	GCCACATCCAACCATGAGCCTT
Acan	Forward	CAGGCTATGAGCAGTGTGATGC
Acan	Reverse	GCTGCTGTCTTTGTCACCCACA
Sfrp1	Forward	CAATACCACGGAAGCCTCTAAGC
Sfrp1	Reverse	GCAAACTCGCTTGCACAGAGATG
Sfrp2	Forward	CCAAGGTGTGTGAAGCCTGCAA
Sfrp2	Reverse	CCAGGATGATCTTGGTGTCTCTG
Sox9	Forward	CACACGTCAAGCGACCCATGAA
Sox9	Reverse	TCTTCTCGCTCTCGTTCAGCAG
Col14A1	Forward	GTCAGGCTTCAGTGATGCTCTG
Col14A1	Reverse	ATTTGCCACCGAGCACACAAGC
Smoc1	Forward	GACTACTGCGACCTGAACAAGG
Smoc1	Reverse	GGTTTGTGCCTGCTCGTTTTCC

Supplementary Table 3: Primer sequences for qPCR of human genes.

Gene (human)	Direction	Primer Sequence (5' to 3')
HPRT	Forward	CATTATGCTGAGGATTTGGAAAGG
HPRT	Reverse	CTTGAGCACACAGAGGGCTACA
SOST	Forward	GGAGCTGGAGAACAACAAGACC
SOST	Reverse	TCACGTAGCGGGTGAAGTGCAG
PIEZO1	Forward	CCTGGAGAAGACTGACGGCTAC
PIEZO1	Reverse	ATGCTCCTTGGATGGTGAGTCC
HES1	Forward	GGAAATGACAGTGAAGCACCTCC
HES1	Reverse	GAAGCGGGTCACCTCGTTCATG
WNT16	Forward	TCGGAAACACCACGGGCAAAGA
WNT16	Reverse	GCGGCAGTCTACTGACATCAAC
TNFRSF11B (OPG)	Forward	GGTCTCCTGCTAACTCAGAAAGG
TNFRSF11B (OPG)	Reverse	CAGCAAACCTGAAGAATGCCTCC
TNFRSF11 (RANKL)	Forward	GCCTTTCAAGGAGCTGTGCAAAA
TNFRSF11 (RANKL)	Reverse	GAGCAAAAGGCTGAGCTTCAAGC
ACAN	Forward	GGAGACAGAGGGACACGTCA
ACAN	Reverse	AGGTGGCTCCATTCAGACAAG
SFRP1	Forward	CAATGCCACCGAAGCCTCCAAG
SFRP1	Reverse	CAAACTCGCTGGCACAGAGATG
SFRP2	Forward	CTCCAAAGGTATGTGAAGCCTGC
SFRP2	Reverse	CCAGGATGATTTTGGTATCTCGG
SOX9	Forward	AGGAAGCTCGCGGACCAGTAC
SOX9	Reverse	GGTGGTCCTTCTTGTGCTGCAC
COL14A1	Forward	CACAAACCTCCTCAGCGGAATG
COL14A1	Reverse	GGCTTGGAGATTGGTAACACCC
SMOC1	Forward	TGATGCCCAGTTGTGAGAGCGA
SMOC1	Reverse	TGTCAGTGGTGAGAGCATCCAG

Supplementary Table 4: Primer sequences for CUT & RUN assay.

Primer ID	Direction	Primer Sequence (5' to 3')
**CUT&RUN Primer-1**	Forward	CCGTGCTTTCCACTCGGT
**CUT&RUN Primer-1**	Reverse	CGCATTCCAGAGGCGTGA
**CUT&RUN Primer-2**	Forward	AACTCACGGCGGCTCG
**CUT&RUN Primer-2**	Reverse	CGAGCTTATAAAGGCCCGCA
