# Supplementary material for: The pivotal role of the Hes1/Piezo1 pathway in the pathophysiology of glucocorticoid-induced osteoporosis
Source: JCI Insight. 2024 Dec 6;9(23):e179963. doi: 10.1172/jci.insight.179963 (PMC11623955; doi:10.1172/jci.insight.179963)

Figure 1I (Piezo1)

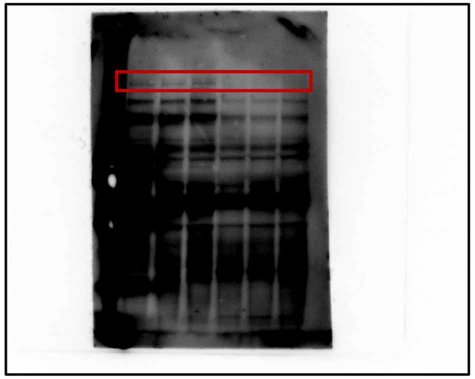

Figure 1I (β-Actin)

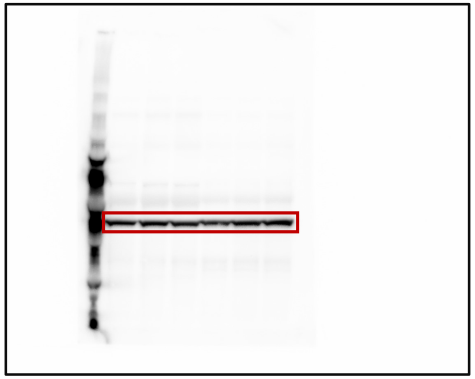

Figure 5E (Piezo1)

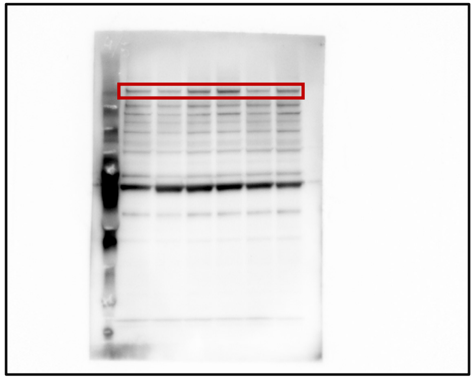

Figure 5E (β-Actin)

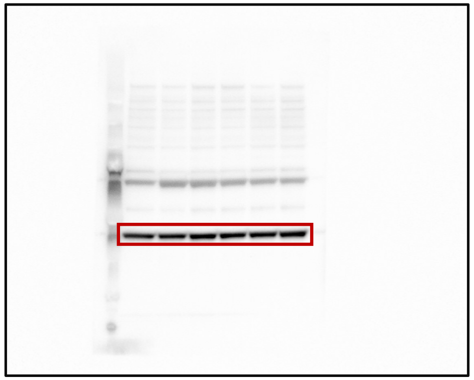

Figure 5F (pAkt)

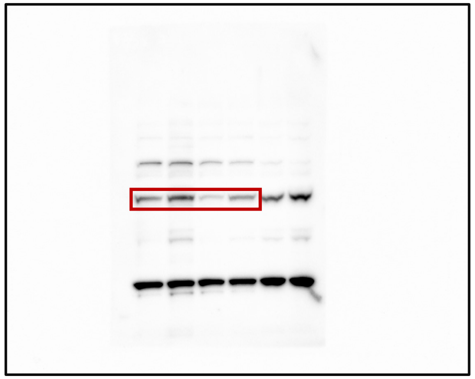

Figure 5F (Akt)

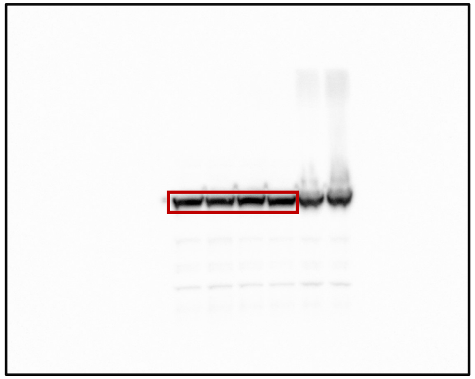

Figure 5F (β-Actin)

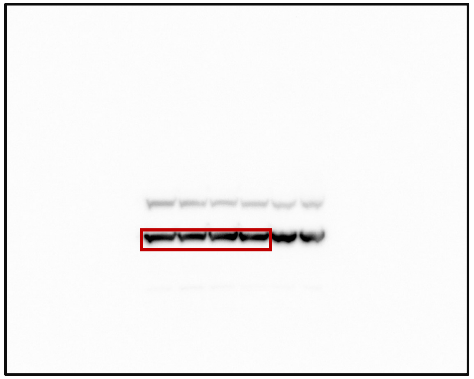

Figure 5G (pERK)

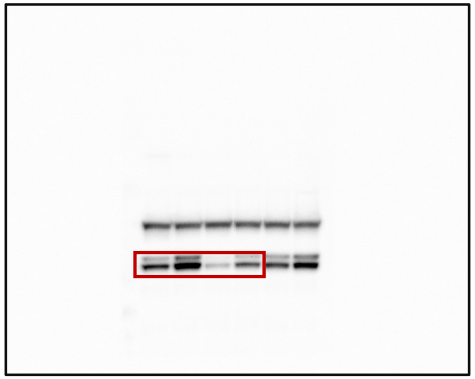

Figure 5G (ERK)

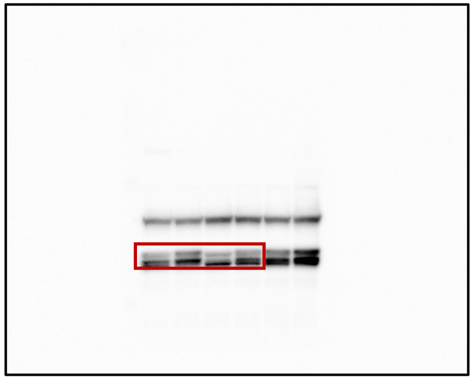

Figure 5G (β-Actin)

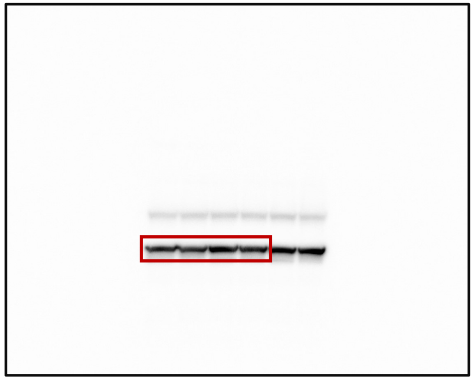

Figure 5J (pAkt)

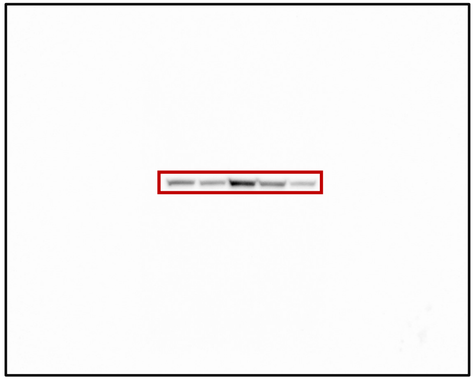

Figure 5J (Akt)

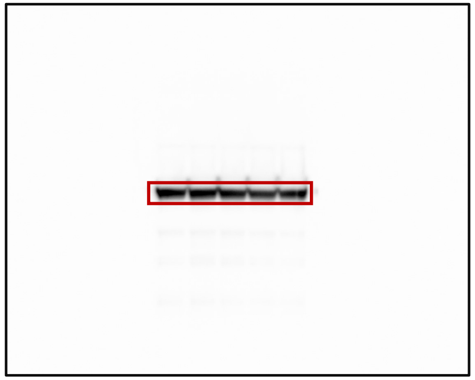

Figure 5J (β-Actin)

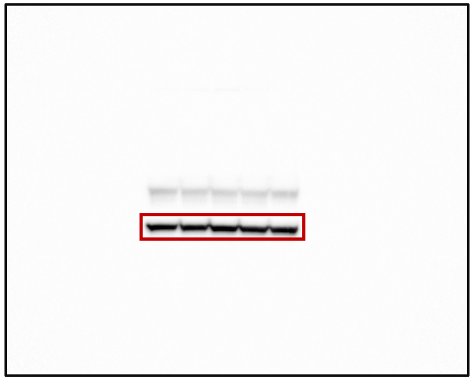

Figure 7B (Hes1)

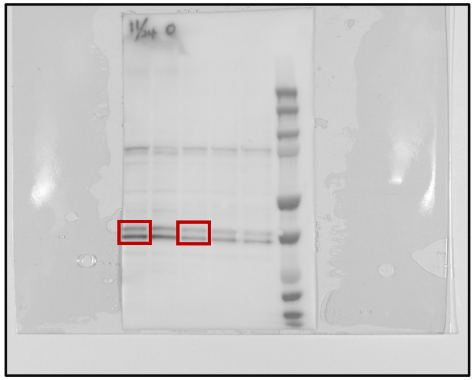

Figure 7B (β-Actin)

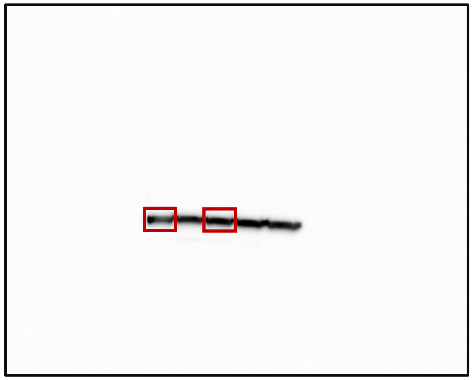

Figure 7B (Piezo1)

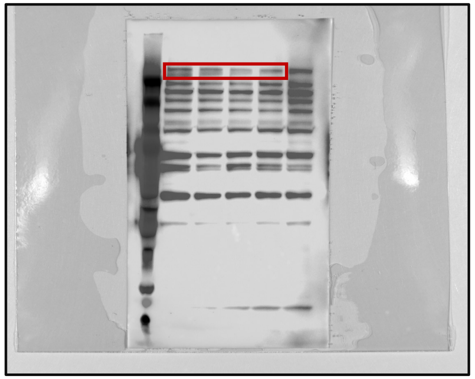

Figure 7B (β-Actin)

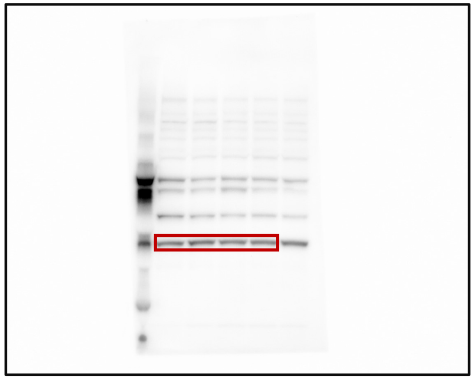

Figure 7H (pHes1)

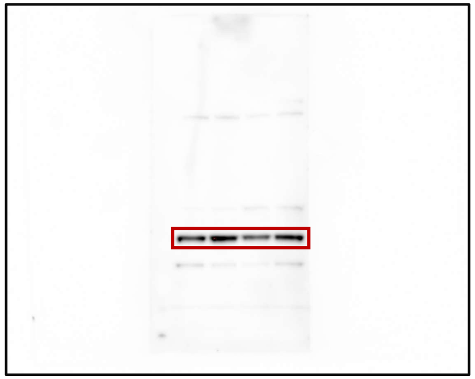

Figure 7H (Hes1)

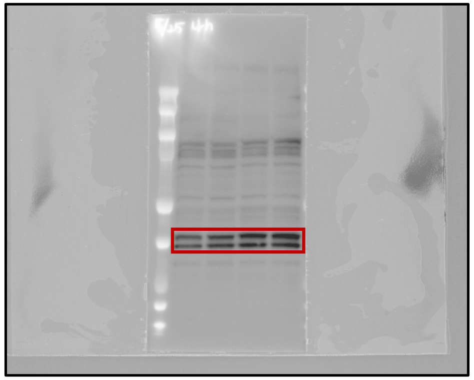

Figure 7H (β-Actin)

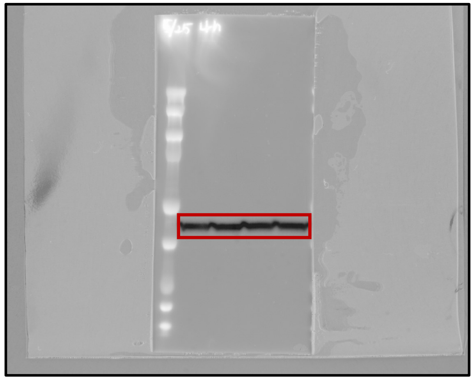

Figure 9A (Piezo1)

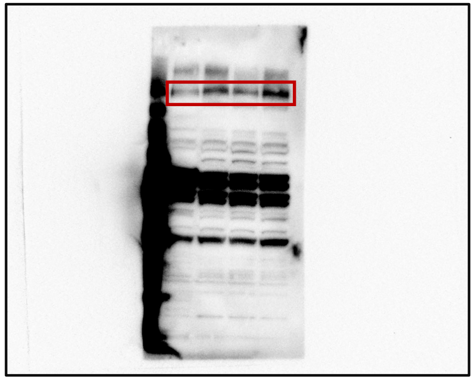

Figure 9A (β-Actin)

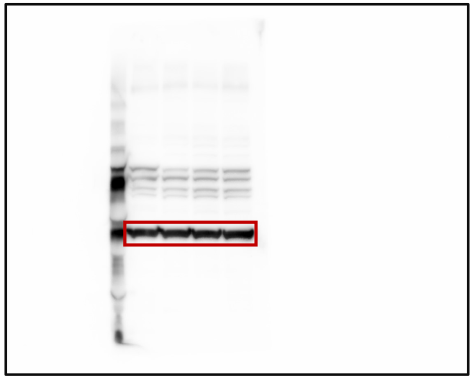

Supplementary Figure 10 (pCaMKII)

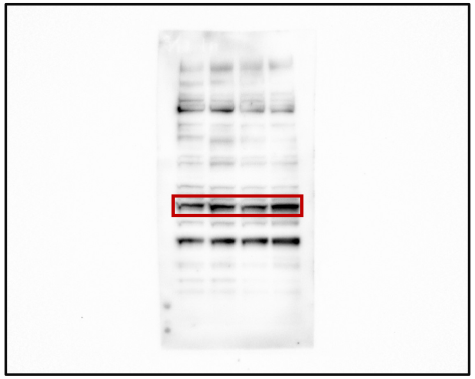

Supplementary Figure 10 (CaMKII)

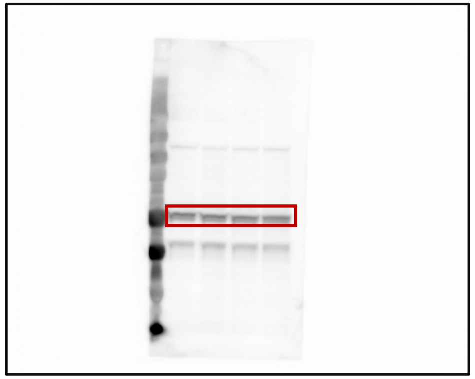

Supplementary Figure 10 (β-Actin)

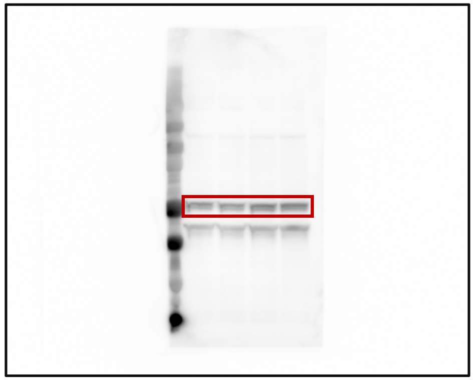

Supplementary Figure 14 (Cleaved Notch1)

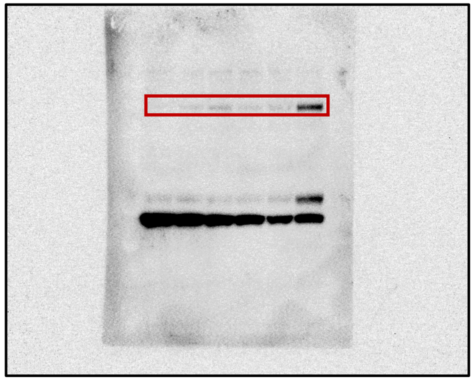

Supplementary Figure 14 (β-Actin)

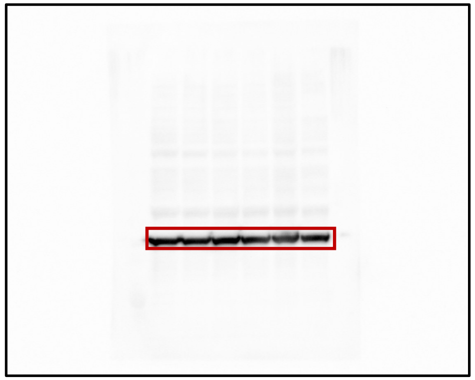

Supplementary Figure 16 (Piezo1)

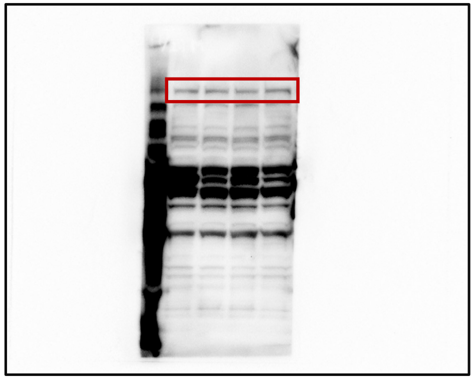

Supplementary Figure 16 (β-Actin)

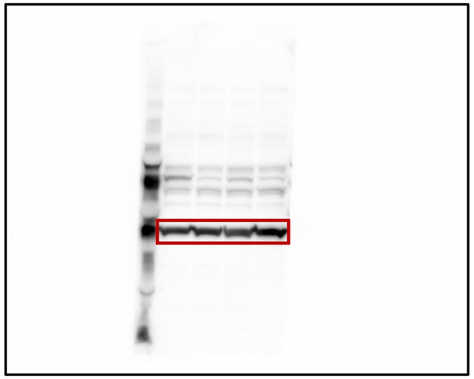

Supplement: Unedited blot and gel images [file jciinsight-9-179963-s040.pdf]
